# Association mapping for agronomic traits in six-rowed spring barley from the USA harvested in Kazakhstan

**DOI:** 10.1371/journal.pone.0221064

**Published:** 2019-08-12

**Authors:** Shyryn Almerekova, Burabai Sariev, Aigul Abugalieva, Vladimir Chudinov, Grigoriy Sereda, Laura Tokhetova, Anarbai Ortaev, Vladimir Tsygankov, Thomas Blake, Shiaoman Chao, Yuliya Genievskaya, Saule Abugalieva, Yerlan Turuspekov

**Affiliations:** 1 Institute of Plant Biology and Biotechnology, Almaty, Kazakhstan; 2 Kazakh Research Institute of Agriculture and Plant Industry, Almalybak, Almaty region, Kazakhstan; 3 Karabalyk Breeding Station, Nauchnyi, Kostanai region, Kazakhstan; 4 Karaganda Breeding Station, Tsentralnoe, Karaganda region, Kazakhstan; 5 Kazakh Research Institute of Rice, Kyzylorda, Kazakhstan; 6 Krasnovodopad Breeding Station, Sarkyrama, Turkestan region, Kazakhstan; 7 Aktobe Breeding Station, Aktobe, Aktobe region, Kazakhstan; 8 Department of Plant Sciences and Plant Pathology, Montana State University, Bozeman, MT, United States of America; 9 USDA-ARS Biosciences Research Lab, Fargo, ND, United States of America; 10 Al-Farabi Kazakh National University, Department of Biodiversity and Bioresources, Almaty, Kazakhstan; University of Helsinki, FINLAND

## Abstract

In barley, six-rowed barley is advantageous over two-rowed barley for feed due to the larger number of seeds per spike and the higher seed protein content. The growth of six-rowed barley is potentially important for breeding in agriculturally oriented countries, such as Kazakhstan. Nevertheless, until recently, very little attention was given to six-rowed barley in breeding projects in Kazakhstan, one of the largest countries in the world. In this study, phenotyping and single nucleotide polymorphism (SNP) genotyping data were generated from 275 accessions originating from six different breeding organizations in the USA as well as 9 accessions from Kazakhstan in field trials at six breeding institutions. The USA six-rowed barley was tested in comparison to local accessions over three years (2009–2011) based on analyses of key agronomic traits. It was determined that the average yield in the USA accessions in comparison to local lines showed heavier yield in all six tested sites. Principal Coordinate Analysis based on 1618 polymorphic SNP markers separated Kazakh lines from six USA barley origin groups based on PC1 (77.9%), and Montana lines from the remaining five USA groups based on PC2 (15.1%). A genome-wide association study based on eighteen field trials allowed the identification of 47 stable marker-trait associations (MTA) for ten agronomic traits, including key yield related characters such as yield per square meter, thousand grain weight, number of kernels per spike, and productive tillers. The comparison of chromosomal positions of identified MTA with positions of known genes and quantitative trait loci suggests that 25 out of those 47 MTAs are presumably novel. The analysis of 42 SNPs associated with 47 MTAs in the Ensemble genome annotation system (http://ensemblgenomes.org) suggested that 40 SNPs were in genic positions of the genome, as their sequences successfully aligned with corresponding Gen ID.

## Introduction

In barley, two-rowed (TR) and six-rowed (SR) germplasm groups can be separated based on the arrangement of triplets at rachis nodes [1]. In SR barley, all three florets of the triplet are fertile, in TR barley lateral spikelets are sterile, and therefore, only one floret is fertile per rachis node. The recessive allele of the *Vrs1* gene is responsible for the SR spike in barley, since the loss of function of *Vrs1* results in fully developed fertile spikelets with SR phenotype [2]. TR and SR types show very different growth habits and expose a pleiotropic effect on a number of yield components [2,3,4] and grain quality [5, 6]. TR barley is cultivated predominantly for the malting industry in most regions of the world, except for the USA and Mexico, where SR barley is also used for this purpose [7].

In Central Asia, the largest barley producer is Kazakhstan, which grows over 80% of the region’s barley [8]. The main objective in barley breeding is the development of new cultivars for feed, as the malting industry is largely undeveloped in this country. Until recently the country was cultivating predominantly TR barley [9], and SR barley was mainly out of scope for local breeders, even though SR barley is advantageous over TR for feeding due to the larger number of seeds per spike and higher protein content in seeds [7]. The introduction of SR barley in this country may have a great impact on the local industry as this may meet the agricultural requirements caused by a shortage of fodder crops for domestic animals. Therefore, an appropriate breeding project with the usage of foreign germplasm resources should be developed in a short period. Countries with similar environments to Kazakhstan in terms of climate and latitude, such as the USA, are potential sources of germplasm for SR barley activities. This approach to the selection of foreign germplasm is particularly interesting, as the USA barley-oriented research organizations have a long history for breeding SR barley [1, 7]. Also, the USA barley cooperative agricultural program (CAP) has generated large barley resources, including germplasm [10, 11] that are available for the development of breeding projects around the World.

Currently, the efficiency of breeding programs might be improved by the application of modern genomic technologies [12, 13], such as the automated genome-wide profiling of large collections based on the use of single nucleotide polymorphism (SNP) markers [14, 15, 16]. In barley, an Illumina-based SNP genotyping platform was extensively used for both the evaluation of wild [17, 18, 19] and cultivated barley accessions [20, 21, 22, 23]. The fast generation of large amounts of SNP genotyping data was particularly successful for the genetic mapping of quantitative trait loci (QTL) of agronomic traits based on genome-wide association study (GWAS). The number of successful publications demonstrating the high efficiency of GWAS in the identification of marker-trait associations (MTA) has grown rapidly in recent years [24, 25, 26, 27, 28].

The other conclusion drawn from the survey of GWAS articles for cereal crops, including barley, suggests a strong influence of environmental conditions on detection of QTL for yield components [29, 30, 31]. Thus, the success in the discovery of novel and important QTL can be achieved in GWAS using SR barley tested in new environmental niches, which in turn may help improve local breeding projects around the world. The main goal of this work was GWAS using SR barley from the USA for the identification of MTA in field trials in six diverse environments of Kazakhstan. The study may help in the detection of promising new SR lines that are well adapted to Kazakhstan, identify new QTL for agronomic traits, and enhance the efficiency of local projects in the country.

## Materials and methods

### Collections of the US six-rowed barley

The spring six-rowed barley panel consists of 275 accessions, which originated from six different breeding organizations in the USA and 9 accessions from Kazakhstan (S1 Table). The US accessions (S1 Table) are part of the USDA barley program [15] and obtained from Dr. T. Blake (Montana State University, MT, USA). The US lines are from Montana State University (MT, 3 lines), Washington State University (WA, 22 lines), Utah State University (UT, 84 lines), University of Minnesota (MN, 92 lines), the Small Cereal Collection of the USDA held in Aberdeen, Idaho (AB, 45 lines), and one private company, Busch Agricultural Resources, a division of the Anheuser-Busch Corporation (BA, 29 lines). Nine barley accessions from Kazakhstan were received from the Kazakh State Seed Testing Organization. The whole collection was studied in the field conditions of six breeding organizations of the country in 2009–2011. Selected breeding organizations represented major barley growing areas in the country and located in Aktobe, Almaty, Karaganda, Kostanai, Kyzylorda, and South-Kazakhstan regions (hereafter abbreviated as AK, AL, KA, KB, KO, and KV, respectively).

### Phenotyping data

In all studied locations except KO, the plants were grown in non-irrigated conditions. The locations of six field trial sites and their geographical, meteorological, and soil type data are given in Genievskaya et al., 2018 [23], as TR and SR types were grown side-by-side in the same fields and years. The collection was planted at each site in randomized experiments, each with three replicates, from 2009 to 2011. The distance between rows was 15 cm, and the distance between plants within a row was 5 cm. The experiments in all regions were conducted in one-metre blocks, except in KO where the accessions were planted in 3 rows per repetition. After collection, the data for mean values of ten agronomic traits of the 285 six-rowed barley accessions were subjected to further statistical analysis. The ten traits included the following: days to heading time (HT), days to seed maturity (SMT), plant height (PH), peduncle length (PL), productive tillering (PT), number of kernels per spike (NKS), spike length (SL), rachis internode length (RIL), thousand grain weight (TGW), and yield per square meter (YM2).

### Genotyping data

Nine accessions from Kazakhstan were genotyped using the GoldenGate Illumina 9K SNP chip at the TraitGenetics company (TraitGenetics GmbH, Gatersleben, Germany). The SNP genotyping data of the USA accessions consisted of 3072 SNP markers [14] and were provided by Dr. T. Blake (MSU, Bozeman, MT, USA) and Dr. S. Chao (USDA-ARS Biosciences Research Lab, Fargo, ND, USA). The polymorphic information content (PIC) values were estimated using DnaSP v6 [32]. Phylogenetic relationship between the USA and Kazakhstan sets of SR barley was assessed by the principal coordinate analysis (PCoA) using GenAlEx 6.5 [33].

### GWAS

As the number of samples from Kazakhstan was insignificant, the GWAS was performed using only the USA set of SR barley. The SNP dataset was filtered using a 10% cutoff for missing data and markers with minor allele frequency ≥ 0.05 were considered for GWAS. Numbers of hypothetical groups ranging from k = 1 to 10 were assessed using 50,000 burn-in iterations followed by 100,000 recorded Markov-Chain iterations. The output from STRUCTURE [34, 35] was analyzed for delta K value (ΔK) in STRUCTURE HARVESTER [36]. Based on the final *k* values, Q-matrix for four identified clusters was developed. GWAS for the above-listed agronomic traits evaluated in six regions was performed using polymorphic SNPs, and implemented in the TASSEL 5 package [37] and MLM test [38, 39]. A minimal threshold bar for MTA was P<10E-4. The genetic map was constructed using MapChart software [40].

### Statistical analysis

Statistical analyses of data, including multiple factor ANOVA, Pearson’s correlation and *t-test* were calculated using the software package GraphPad Prism 5.0 (GraphPad Software, La Jolla California USA. www.graphpad.com). GGE Biplot methods were employed by using the GenStat package (17^th^ release, VSN International, Hertfordshire, UK). The symmetric scaling option of both methods and available field data for all three sites were used in estimations.

## Results

### Field performance of US six-rowed barley

The Pearson correlation coefficient indicated a large environmental influence on barley performance in different locations, as averaged YM2 in six locations over three years was correlated only in three out of fifteen cases. In two of those three cases, the KB station showed the correlations with the KO and KA sites, while the KA site was highly correlated with the AK (Table 1).

**Table 1:** Pearson correlation index for averaged YM2 in six field stations.

	KO	KV	KA	KB	AK
KO	.	.	.	.	.
KV	-0.028	.	.	.	.
KA	0.011	0.041	.	.	.
KB	0.136*	-0.040	0.134*	.	.
AK	-0.101	0.033	0.175**	0.062	.
AL	-0.043	0.094	0.019	0.003	0.002

*–P<0.05

In the YM2 analysis, the three-way ANOVA confirmed that the environment (E) plays a significant role, as Region and Year of the study were the only factors that significantly contributed to the yield performance (Table 1). However, the genotype (G) also showed significant importance in genotype-environment interaction (GEI) as the origin of the accessions played an important role for three agronomic traits related to the spike architecture (NKS and TGW), and contributed to the PH (Table 2).

**Table 2:** Three-way ANOVA for the collection of SR barley tested at six environments by seven agronomic traits.

Factors	HT	SMT	PH	PT	TGW	NKS	YM2
**Origin**	1.977	0.030	5.991*	0.126	40.9***	8.0**	2.158
**Region**	3917.2***	3588.1***	737.8***	4129.4***	1263.1***	300.7***	1809.668***
**Year**	1314.8***	2.587	3.612	284.5***	841.7***	822.9***	2716.148***
**Origin:Region**	1.957	1.885	1.275	0.232	4.307	0.614	1.818
**Origin:Year**	1.517	1.406	0.005	1.154	2.686	7.4**	1.031
**Region:Year**	86.3***	768.1***	220.4***	742.1***	203.2***	200.3***	517.929***
**Origin:Region:Year**	5.2***	2.6*	0,344	1,906	6.9***	3.1**	1.770

**Fig 1:**
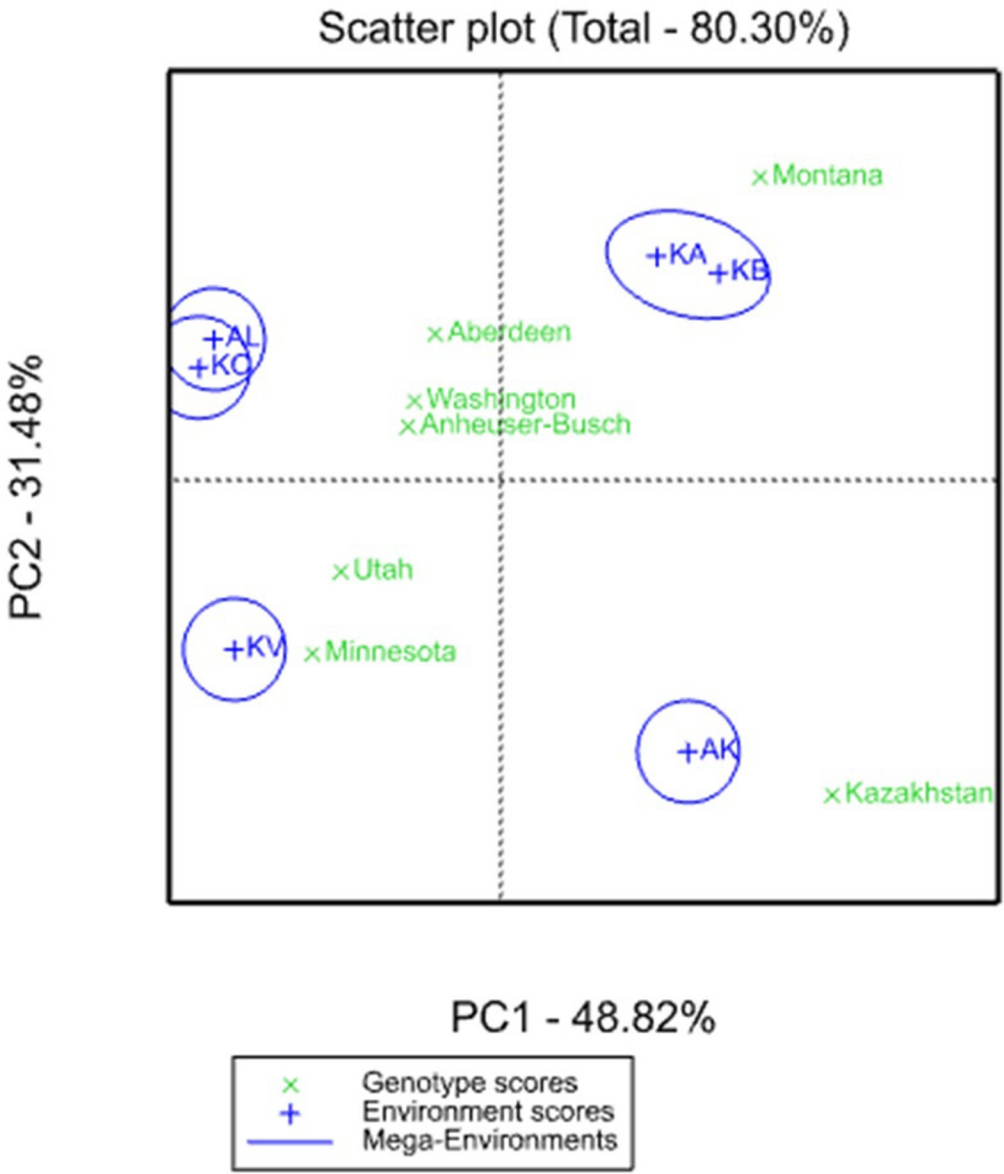
GGE bilot of six-rowed barley accessions from the USA and Kazakhstan studied at six field regional breeding organizations of Kazakhstan. AK–Aktobe, AL–Almaty, KA–Karaganda, KB–Kostanai, KV–South Kazakhstan, KO–Kyzylorda.

The GGE biplot graph assessment suggests that six tested environments can be divided to four major regions, as conditions of barley growth in KA and KB, as well as AL and KO, were grouped together, and formed opposite mega-environments in the upper part of the GGE biplot graph (Fig 1). As genotype score of KZ lines landed near AK site, it seems local accessions were well suited for the western part of the country. The US accessions were suited for the remaining three mega-environments, as MT lines well matched to KA and KB sites, AB, WA, and AN lines to AL and KO sites, and UT and MI lines to KV site (Fig 1).

**Fig 2:**
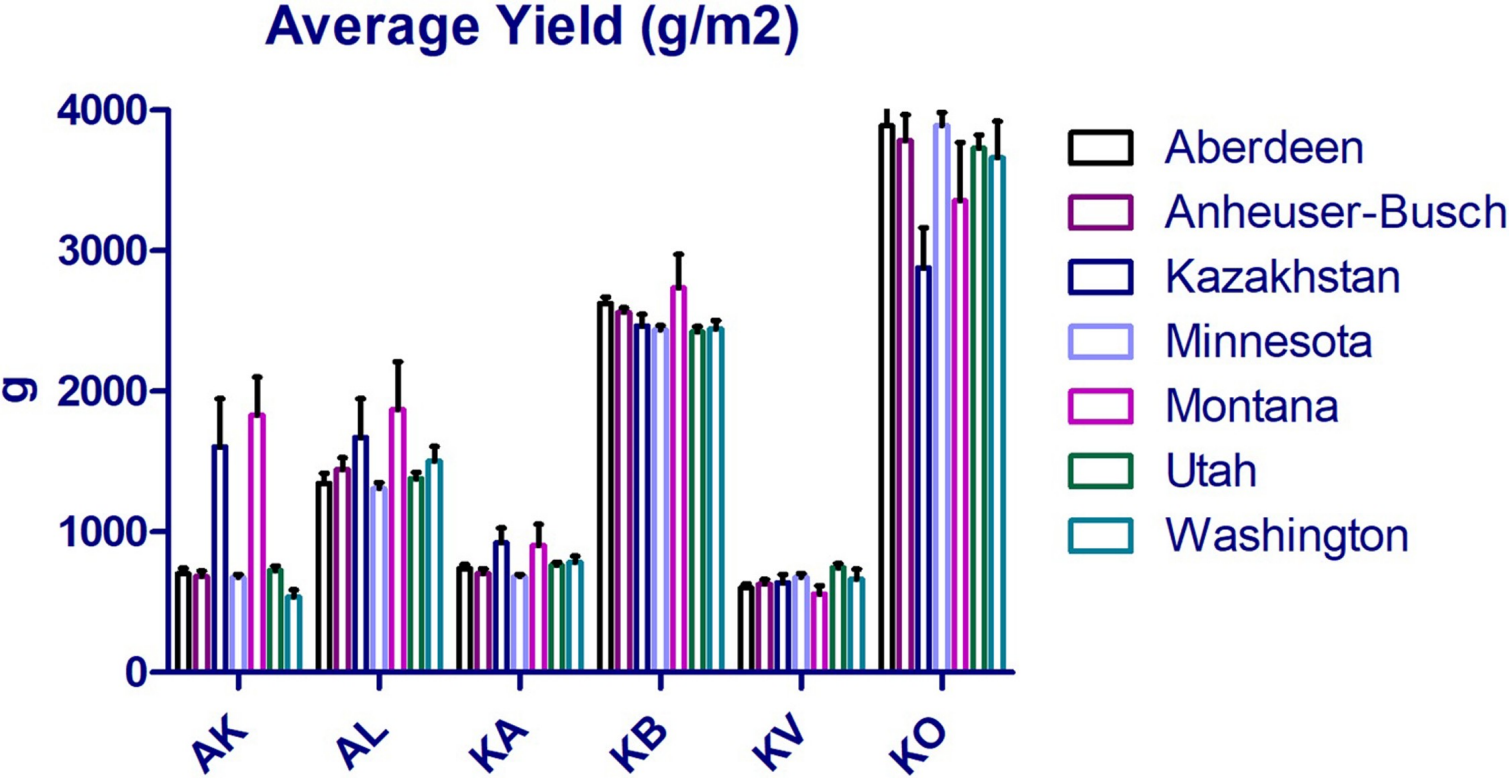
Comparison of average yield per meter among six-rowed barley originated in breeding institutions in the USA and Kazakhstan at six regional agricultural organizations in Kazakhstan. AK–Aktobe, AL–Almaty, KA–Karaganda, KB–Kostanai, KV–South Kazakhstan, KO–Kyzylorda.

US SR accessions showed an outstanding field performance in comparison to local cultivars and breeding lines in irrigated plots of Kyzylorda region (Fig 2). Three MT accessions showed insignificant YM2 advantages at the three locations, AK, AL, and KB (Fig 2). As the KO station was the only irrigated site among studied locations, the average YM2 value over three years (2009–2011) was the highest (3598.3 ± 211.2 g) in this southern region of the country (Fig 2). Among non-irrigated sites, the highest average yield was recorded in KB station (2527.7 ± 76.3 g), located in Northern Kazakhstan (Fig 2), where the country has more than 80% of total barley growing area. Therefore, particular attention concerning the yield performance of the studied collection was given to the analyses of different US barley origins harvested in the KB station.

The Pearson index analysis of the field data from the KB site suggested that yield is positively correlated to SMT, PH, and TGW (Table 3). Interestingly, the results in Table 3 suggested that heavier YM2 is related to slower seed maturation time and taller plant height. The correlation between YM2 and HT was negative, although the index value was non-significant (Table 3).

**Table 3:** Pearson correlation index between traits of six-rowed barley at the KB site based on averaged data over three years of trials.

	HT	SMT	PH	PT	NKS	YM2
HT	.	.	.	.	.	.
SMT	-0.562***	.	.	.	.	.
PH	0.002	0.127*	.	.	.	.
PT	-0.038	0.015	0.116*	.	.	.
NKS	0.165**	0.069	0.199**	-0.111	.	.
YM2	-0.092	0.177**	0.187**	0.016	0.089	.
TGW	-0.096	0.135*	0.216**	0.004	-0.203**	0.272***

*–P<0.05

**–P<0.001

***–P<0.0001

At the KB site, the highest averaged YM2 over three years was recorded for MT (2734.7 g/m) lines, followed by AB (2625.9 g/m) and BA (2562.5 g/m) accessions. In total, 28 US accessions showed better yield performance over the best local breeding line L50/T26 (2872.1 g/m). The highest averaged YM2 among individual accessions was scored by line 2627 (3778.6 g/m) originated in Utah (S2 Table).

### Genetic diversity and population structure analyses

The genotyping of USA accessions allowed the identification of 1618 common polymorphic SNP markers distributed over all seven chromosomes with an average spacing of 0.704 cM. The number of SNP per chromosome ranged from 191 on chromosomes 1H and 4H to 260 on chromosome 3H. The data also included 62 SNPs with unknown (U) positions. Additional information for each individual U marker can be retrieved from Muñoz-Amatriaín et al., 2014 [15] and the physical map Morex x Barke, 2016 [41]. The chromosomal length varied from 123.29 cM in chromosome 4H to 196.85 cM in chromosome 5H, with the average distance being 155.52 cM per chromosome. Polymorphism information content (PIC) values varied between 0.28 (2H) and 0.34 (3H).

**Fig 3:**
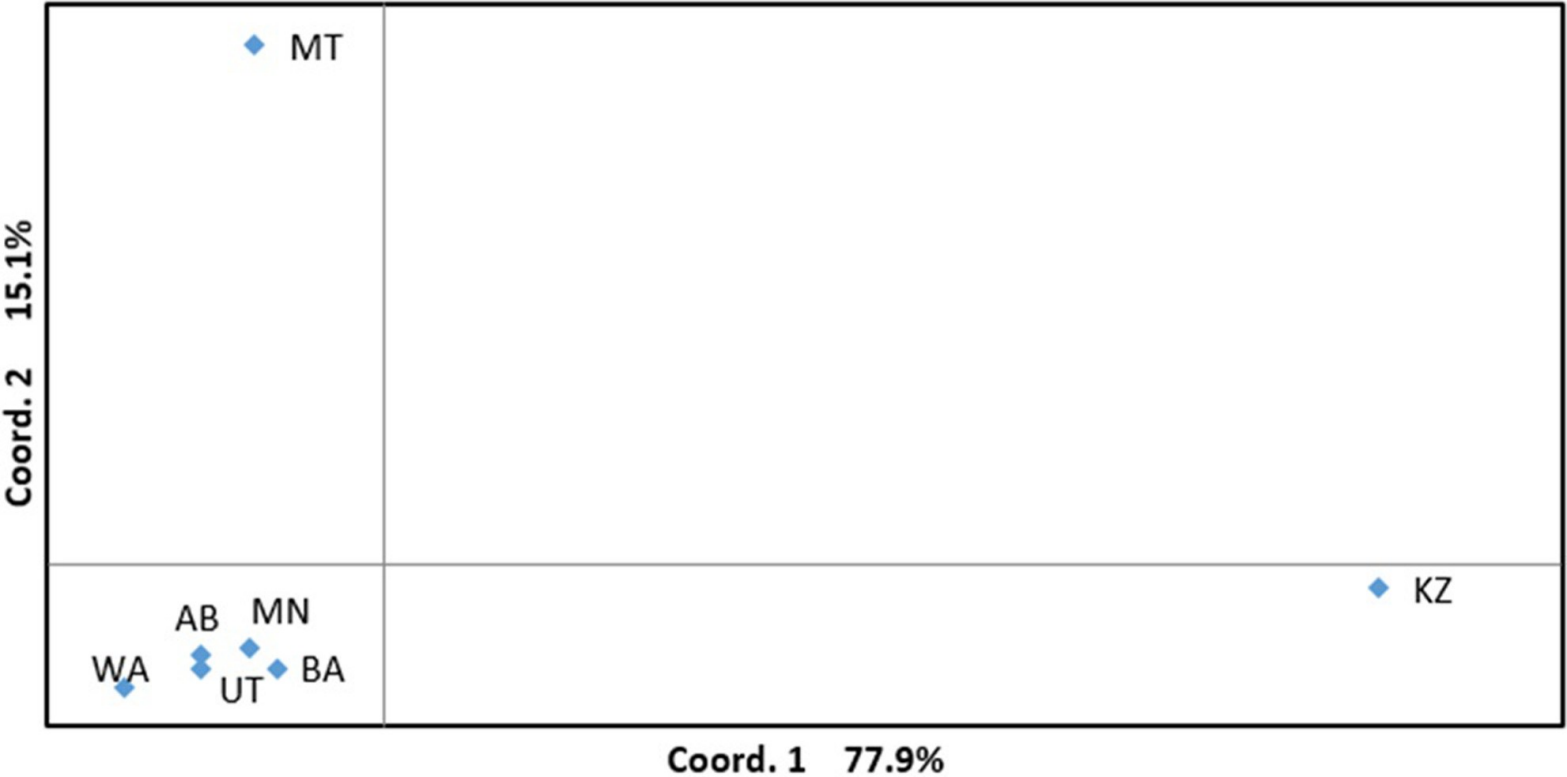
Principal coordinate analysis of SR barley accessions based on SNP data. AB–Aberdeen, BA–Busch Agricultural Resources, MN–the University of Minnesota,—MT–Montana State University, WA–Washington State University, UT–Utah State University, KZ- Kazakhstan.

PCoA based on the analysis of 1618 polymorphic SNP markers was applied to assess the genetic relationship of the USA accessions from six different breeding organizations and Kazakh samples. The PCoA graph suggested that Kazakhs samples were well separated from the USA accessions (Fig 3). As PC1 clearly split Kazakh and USA samples, PC2 separated MT lines from lines of the remaining five US breeding organizations. The plot indicates that six-rowed breeding lines of these five US breeding organizations were genetically clustered together on the left-bottom part of the graph.

### Identification of marker-trait associations based on GWAS

**Fig 4:**
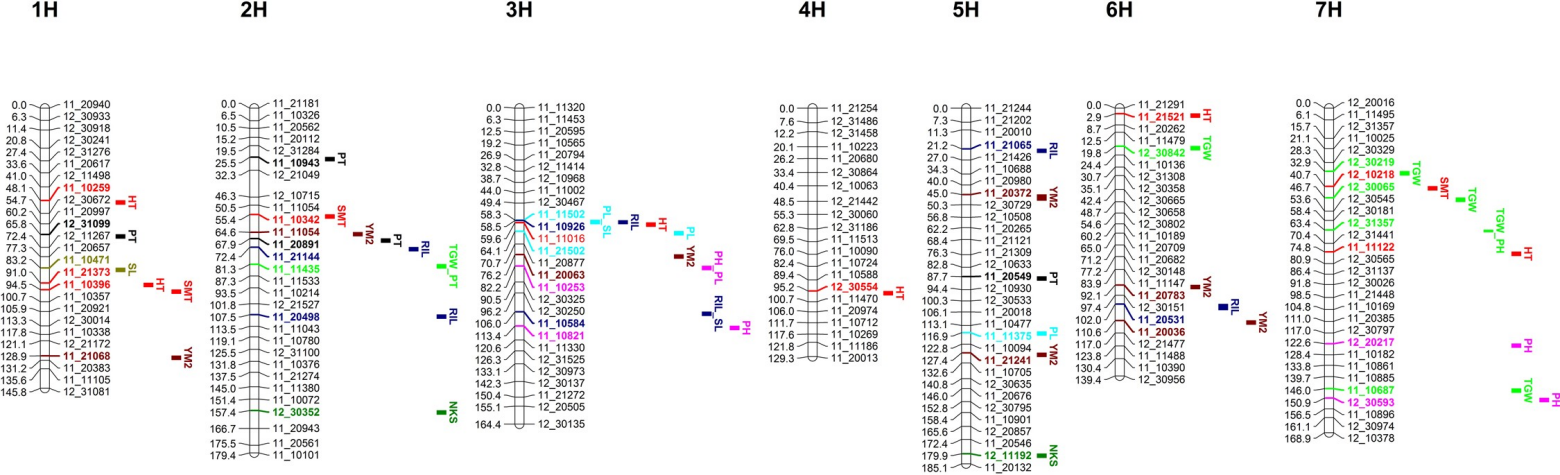
Location of identified MTA on seven barley chromosomes (1H-7H). SNPs and abbreviations of traits are given on the right, and positions of SNPs are shown in cM on the left. HT–days to heading, SMT–days to to seed maturation, PH–plant height, PL–peduncle length, PT–productive tillering, SL–spike length, NKS–number of kernels per spike, RIL–rachis internode length, TGW–thousand kernel weight, YM2 –yield per square meter.

The distribution lines of the QQ plots (S1 Appendix) for ten studied traits indicated the successful correction of the analysis due to the use of both K and Q matrices. At the first stage of the GWAS, 218 MTA were identified for ten studied traits in 18 field trials (6 environments x 3 years) by using the criteria P<10E-4. However, only 47 MTA were statistically significant in two or more environments for studied traits (Table 4 and Fig 4), and only those relatively stable MTA were further evaluated (Table 4). Among six environments, the highest number of MTA were identified in KB (n = 20), followed by KA (n = 15), and AL (n = 13). Identified 47 MTA were linked to 42 SNP markers (Fig 4), as only 5 SNPs were involved in MTA with two traits simultaneously. The largest number of SNPs associated with identified MTA were located on chromosome 7H (10 SNPs), followed by chromosome 2H (9 SNPs), and 3H (8 SNPs).

**Table 4:** List of identified SNPs for ten studied traits in six-rowed barley collection.

Trait	Marker ID	Chr.	Pos.(cM)	Alleles	MAF	R^2^ (%)	Max. P-value	Env.*
HT	11_10259	1H	48,12	G/A	0,051	0,117	1,683E-07	3
HT	11_21373	1H	90,98	G/A	0,085	0,083	8,358E-06	6
HT	11_11016	3H	59,56	G/C	0,153	0,049	9,556E-04	2
HT	12_30554	4H	95,23	C/A	0,249	0,049	2,878E-04	3
HT	11_21521	6H	2,86	G/C	0,259	0,050	3,733E-04	3
HT	11_11122	7H	74,81	A/G	0,087	0,051	5,077E-04	2
SMT	11_10396	1H	94,48	C/G	0,096	0,054	1,493E-04	2
SMT	11_10342	2H	55,41	G/A	0,086	0,056	1,804E-04	2
SMT	12_10218	7H	40,68	C/G	0,267	0,054	1,597E-04	2
NKS	12_30352	2H	157,42	G/A	0,137	0,072	8,992E-06	2
NKS	12_11192	5H	179,86	A/G	0,101	0,047	3,146E-04	2
PH	11_10253	3H	82,19	G/A	0,296	0,071	7,490E-05	4
PH	11_10821	3H	113,39	A/G	0,054	0,061	1,117E-04	2
PH	12_31357	7H	63,4	A/G	0,068	0,043	6,953E-04	2
PH	12_20217	7H	122,6	T/A	0,050	0,047	4,163E-04	2
PH	12_30593	7H	150,86	A/G	0,152	0,045	5,827E-04	2
PL	11_11502	3H	58,31	C/A	0,229	0,067	4,190E-05	2
PL	11_21502	3H	64,07	G/A	0,186	0,053	4,829E-04	2
PL	11_10253	3H	82,19	G/A	0,296	0,061	2,068E-04	2
PL	11_11375	5H	116,93	A/G	0,124	0,050	6,854E-04	2
PT	12_31099	1H	65.8	G/A	0,084	0,069	2,898E-05	2
PT	11_10943	2H	25,53	G/C	0,165	0,046	7,143E-04	2
PT	11_20891	2H	67,89	A/G	0,051	0,080	1,047E-05	2
PT	11_11435	2H	81,26	A/G	0,057	0,058	1,698E-04	2
PT	11_20549	5H	87,71	C/A	0,076	0,062	1,872E-04	2
RIL	11_21144	2H	72,44	A/G	0,081	0,053	2,525E-04	2
RIL	11_20498	2H	107,47	G/A	0,101	0,040	7,182E-04	2
RIL	11_10926	3H	58,31	T/A	0,282	0,055	1,483E-04	2
RIL	11_10584	3H	105,98	A/T	0,172	0,104	1,585E-04	3
RIL	11_21065	5H	21,24	A/G	0,194	0,049	2,859E-04	3
RIL	11_20531	6H	102,03	G/A	0,060	0,075	1,129E-05	3
SL	11_10471	1H	83,15	G/A	0,064	0,045	6,953E-04	2
SL	11_11502	3H	58,31	C/A	0,229	0,052	3,253E-04	2
SL	11_10584	3H	106,98	A/T	0,172	0,069	3,230E-05	2
TGW	11_11435	2H	81,26	A/G	0,057	0,060	3,250E-04	2
TGW	12_30842	6H	19,8	G/A	0,300	0,042	8,017E-04	2
TGW	12_30219	7H	32,88	G/A	0,239	0,044	6,497E-04	2
TGW	12_30065	7H	46,74	G/A	0,255	0,048	3,647E-04	2
TGW	12_31357	7H	63.4	A/G	0,068	0,051	2,542E-04	2
TGW	11_10687	7H	146,03	C/A	0,054	0,079	2,583E-05	3
YM2	11_21068	1H	128,92	G/A	0,252	0,044	9,111E-04	2
YM2	11_11054	2H	64,55	A/G	0,094	0,060	2,015E-04	2
YM2	11_20063	3H	76,17	A/G	0,270	0,044	8,368E-04	2
YM2	11_20372	5H	44,99	G/A	0,060	0,049	4,100E-04	3
YM2	11_21241	5H	127.43	A/G	0,278	0,052	3,337E-04	2
YM2	11_20783	6H	92,12	G/A	0,154	0,053	3,991E-04	2
YM2	11_20036	6H	110,59	A/G	0,063	0,049	4,794E-04	2

*—Number of environments representing identified MTAs

The analysis of the database of the barley genome in the Ensemble genome annotation system (http://ensemblgenomes.org) suggests that only 2 out of 42 identified SNPs were outside of the genic region (S3 Table). A survey of the literature showed that 22 out of 47 identified MTA in this study were previously reported in referred articles (S3 Table). The GWAS for four plant adaptation traits HT, SMT, PH, and PL allowed the identification of 18 MTA linked to 17 SNPs. In two chromosomal regions (1H and 3H), the distinct SNPs for these traits were positioned next to each other, suggesting that they theoretically may link to a major gene associated with none of those plant adaptation traits. In chromosome 1H, the SNPs 11_21375 and 11_10396 were linked with HT (91.0 cM) and SMT (94.5 cM), respectively. In chromosome 3H, the SNPs located between 58.3 cM and 59.6 cM were associated with PL and HT, respectively.

The largest number of identified MTA were found for YM2 (n = 7) and the number of MTA in other yield-related traits were as following, TGW (n = 6), RIL (n = 6), PT (n = 5), SL (n = 3), and NKS (n = 2) (Table 4 and Fig 4). The most frequently identified MTA was one for HT at the chromosome 1H (91.0 cm), which was significant in 6 different environments (Table 4), followed by MTA for PH at the chromosome 3H (82.2 cM), which was significant in 4 different environments. Among MTA for YM2, the most common was the association at the chromosome 5H (45.0 cM) (Table 4). This MTA was found to be significant in Northern Kazakhstan, which is the most important barley growing are in the country.

## Discussion

### Field performance of the USA six-rowed barley accessions in Kazakhstan

Field trials in six different environments suggested that tested US SR accessions showed an outstanding field performance in comparison to local cultivars and breeding lines, as in all six locations the USA lines prevailed over Kazakh lines (Fig 2). The advantage of the US samples was particularly visible at KO irrigated sites. It was also important to find out that the average YM2 over three years in 28 US accessions outperformed the Kazakh line L50T26 in conditions of Northern Kazakhstan, where the country has over 80% of total barley growing area. L50T26 showed best average yield performance over three years in Northern Kazakhstan and selected for the comparison because of lack of a local standard for six-rowed barley, suggesting that local breeders ignored SR type of barley in local breeding projects. Thus, field performance of the US barley showed remarkable possibilities to incorporate this germplasm into local breeding schemes for higher grain productivity. As the US lines showed comparatively high YM2 in all studied environments (Fig 2), it is highly likely that they may adapt well to breeding sites from southern (KV and KO) to northern (KB) areas of Kazakhstan, the ninth largest country in the world by territory. The assessment of field performance of the US accessions in different locations in Kazakhstan suggests that an SR barley breeding program in Kazakhstan may achieve success, as this type of barley previously was largely neglected by local breeders and farmers.

As the ANOVA revealed a significant contribution of E and GE in GEI for the studied agronomic traits (Table 2), it was important to assess the differences among environments and the relationship between genotypes of six different breeding origins and six studied environments. The application of the GGE biplot method by using the average YM2 has allowed the separation of six different locations to four distinct mega-environments (Fig 1). In addition, the GGE biplot indicated that Kazakh genotypes were suitable for the western region Aktobe, which is a most drought stressful location among studied sites. The US accessions split to three groups, as MT lines performed well in the conditions of KB and KA (northern and central regions), AB, BA, and WA lines were close to south-east and east regions (AL and KO, respectively), and MN and UT lines performed well in the far eastern region of the country (Fig 1). The results suggest that three MT lines are better adapted and advantageous in the northern region, which confirms a report from the study of TR barley [23].

The genetic relationship of SR lines among groups of six different breeding origins based on the use of 1618 polymorphic SNP markers suggested a rather different outcome (Fig 3). The first coordinate in the PCoA separated Kazakh lines from the US lines, while the second coordinate suggested that MT lines were genetically distant from remaining lines of five USA breeding organizations. Interestingly, the PCoA results are very different from a similar study of TR barley [23], as there both WA and MT lines were very close to Kazakh breeding lines. In ST barley, the accessions from five USA breeding organizations were close to each other, except MT lines. In TR barley, UT, AB, and BA were separated from WA and MT lines based on the first coordinate analysis, and UT was separated from AB and BA based on the second coordinate analysis [23]. It is obvious that the differences in PCoA plots reflect discrete end use targets in the breeding projects of SR and TR types in the USA.

### Identification of novel MTA in six-rowed barley accessions

It was hypothesized that study of SR barley might unmask novel QTL locations for yield-related traits since none of them carries dominant alleles of the *Vrs1* gene. Therefore, current GWAS was focused on the analysis of SR barley collection in six different locations in the country. In this study, the total number of MTA was 47 MTA for ten agronomic traits, which is nearly half as much as in similar research using TR barley based GWAS. A previously reported TR barley study scored 91 MTA for nine traits [23]. The comparison between SR and TR based GWAS allows the identification of 38 MTA in SR analyses that were not detected in TR study, as only 9 common MTA were found in the same genetic locations (S3 Table). In addition, a survey of the scientific literature suggested that 25 out of 47 detected MTA were not reported yet (S3 Table). Therefore, it seems that the tested hypothesis for identification of a possible novel candidate QTL in the accessions with non-functional *Vrs1* gene was supported by the experiment. Furthermore, both that 22 out of 47 MTA were reported elsewhere (S3 Table) and the strength of the generated QQ plots (S1 Appendix) indicate the robustness of the conducted GWAS.

Although it is not strict, ten analyzed traits in this study can be divided into three groups. The first group of traits includes HT, SMT, PH, and PL, as they are all related to plant adaption. The second group consists of SL and RIL, as they associated with spike architecture. PT, NKS, TGW, and YM2 form the third group of traits, as they directly influence the yield. The GWAS of the first group of traits allowed the identification of 13 SNPs, including 6 for HT, 3 for SMT, and 4 for PH (Table 4 and Fig 4). In the list of these 13 markers, one SNP for HT (11_21521) and three SNPs for SMT (11_10396, 11_10342, and 12_10218) are possibly candidate DNA markers for a novel QTL (S3 Table). An analysis of publications related to these traits [23, 25, 42, 43, 44, 45] suggests a novel MTA has been identified. The GWAS of the second group of traits for SL and RIL allows the detection of 7 SNPs that link to 6 MTA for RIL and 3 MTA for SL (S3 Table). The SNPs on chromosome 3H 11_11502 (58.3 cM) and 11_10584 (107.0 cM) were associated with both RIL and SL and might be a novel MTA for these traits. Also, a survey of the publications related to identification of the genetic factors that control spike length [22, 46, 47, 48, 49, 50] suggests that three SNPs for RIL (11_20498, 11_21065 and 11_20531) and one SNP for SL (11_10471,) were a candidate DNA markers for a putative MTA related to these two traits (S3 Table). The GWAS for the third group allowed the identification of four MTA for PT, two for NKS, five for TGW, and seven for YM2 (S3 Table). The comparison of SNP positions of the detected MTA with previously reported associations (S3 Table) suggests that GWAS for SR barley has identified novel SNPs for PT (11_10943 and 11_20891), NKS (12_30352 and 12_11192), TGW (12_30842 and 12_30065), and YM2 (11_11054, 11_20372, 11_20783 and 11_20036).

In general, the GWAS in this work was based on a lower statistical threshold, and therefore, the majority of identified MTA were minor ones (Table 4). However, our previous GWAS study using TR barley accessions [23] suggested that lowering the threshold aids in the detection of trustworthy and relevant MTA. The high number of candidates for novel MTA is apparently related to two major factors. Firstly, the lack of accessions with a dominant allele of the *Vrs1* uncovered hidden genetic factors, which are involved in controlling the studied agronomic traits. Secondly, since the ANOVA suggested a large influence of the environmental factors, the variation in agronomic traits can be explained by the sensitivity of the US accessions to new growth conditions at crucial phases of plant developments, such as HT [51, 52]. Therefore, the application of the GWAS in previously unstudied environments may play an additional role in the detection of novel MTA given in Tables 4 and S3. The other implication of the results is that identified MTA in this study can be useful in breeding projects in other important barley growing regions of the world with similar latitude coordinates. The comparative analysis of GWAS for SR barley harvested in the USA and Kazakhstan would be particularly interesting to assess identified MTA in this study.

In general, the results of the GWAS may lead to two promising directions in genetic and breeding of SR barley. One direction provides bases for isolation of new genes controlling important agronomic traits, which may significantly expand the opportunities for genetic engineering in plants. The other direction is that identified SNPs for MTA might be transformed to cost-effective competitive allele-specific PCR (KASP) assays [53] for their application in breeding based on marker-assisted selection approach. In following stages of the study, KASP assays will be validated for efficiency by testing additional genetic panels, including hybrid lines from the crosses of the USA and Kazakhstan lines, and mapping populations available in the local barley community.

## Conclusions

The YM2 analysis suggests that the SR accessions from the USA outperformed local samples in all six studied locations. Obtained results indicated a high potential of the USA lines as valuable breeding material for local barley breeding projects. The field trials of SR barley from the USA at the KB site, which represents northern regions, which in turn are home to over 90% of the barley territory in the country, have allowed the identification of 28 US accessions that displayed better average yield performance than the best local breeding line. Since SR barley that carries non-functional *Vrs1* gene was tested in six regions over three years, it was hypothesized that GWAS might uncover novel MTA for tested agronomic traits. Indeed, the study revealed that 25 out of 47 detected MTA were not previously reported in scientific articles and potentially can be novel associations for key agronomic traits in barley. Four presumably novel MTA were found for YM2, PT, and RIL, three for TGW, two for NKS, SL and SMT, and one MTA for HT and PH. The study shows the advantage of international exchange and evaluation of germplasm for enhancement of breeding projects in different parts of the world.

## Supporting information

S1 Appendix The distribution lines of the quantile-quantile (QQ) plots for six studied traits in six environments. A–AK (Aktobe), B–AL (Almaty), C–KA (Karaganda), D–KB (Kostanai), E–KO (Kyzylorda), F–KV (South Kazakhstan).
(PDF)

S1 Table List of six-rowed spring barley accessions from six breeding programs in the USA and Kazakhstan. (XLS)

S2 Table Average data for ten agronomic traits of six-rowed barley collection at the Karabalyk breeding station (North Kazakhstan) over 2009–2011. (XLSX)

S3 Table Comparison of identified MTA for six-rowed barley in this study to previously reported genetic factors related to ten analyzed traits. (PDF)

## References

[1] Hayes PM, Castro A, Marquez-Cedillo L, Corey A, Henson C, Jones BL, et al Genetic diversity for quantitatively inherited agronomic and malting quality traits In: Von Bothmer R, van Hintum T, Knüpffer H, Sato K, editors. Diversity in barley (Hordeum vulgare). Elsevier;2003 p. 201–226

[2] Komatsuda T, Pourkheirandish M, He C, Azhaguvel P, Kanamori H, Perovic D, et al Six-rowed barley originated from a mutation in a homeodomain-leucine zipper I-class homeobox gene. PNAS. 2007;104(4): 1424–9. 10.1073/pnas.0608580104 17220272 PMC1783110

[3] Allard R. W. Genetic changes associated with the evolution of adaptedness in cultivated plants and their wild progenitors. Journal of Heredity. 1988;79(4):225–238. 10.1093/oxfordjournals.jhered.a110503 3166481

[4] Liller CB, Neuhaus R, Von Korff M, Koornneef M, Van Esse W. Mutations in barley row type genes have pleiotropic effects on shoot branching. PLoS One. 2015;10(10): e0140246 10.1371/journal.pone.0140246 26465604 PMC4605766

[5] Bowman JGP, Blake TK, Surber LMM, Habernicht DK, Bockelman H. Feed-quality variation in the barley core collection of the USDA National Small Grains Collection. Crop Sci. 2001;41(3):863–70. 10.2135/cropsci2001.413863x

[6] Turuspekov Y, Martin JM, Bowman JGP, Beecher BS, Giroux M J. (2008). Associations Between Vrs1 Alleles and Grain Quality Traits in Spring Barley Hordeum vulgare L. Cereal chem. 2008;85(6):817–23. 10.1094/CCHEM-85-6-0817

[7] Riggs TJ, Kirby EJM. Developmental consequences of two-row and six-row ear type in spring barley: 1. Genetical analysis and comparison of mature plant characters. The Journal of Agricultural Science. 1978;91(1):199–205. 10.1017/S0021859600056768

[8] Aw-Hassan A, Shideed K, Ceccarelli S, Erskine W, Grando S, Tutwiler R. The impact of international and national investment in barley germplasm improvement in the developing countries In. Evenson RE, Gollin D, editors. Crop Variety Improvement and Its Effect on Productivity: The Impact of International Agricultural Research. CABI Publishing;2003 p. 242

[9] Turuspekov Y, Sariev B, Chudinov V, Sereda G, Tokhetova L, Ortaev A, et al Genotype×environment interaction patterns for grain yield of spring barley in different regions of Kazakhstan. Russian Journal of Genetics. 2013;49(2):196–205. 10.1134/S1022795413020129 23668088

[10] Blake VC, Kling JG, Hayes PM, Jannink JL, Jillella SR, Lee J, et al The Hordeum toolbox: the barley coordinated agricultural project genotype and phenotype resource. The Plant Genome. 2012;5(2):81–91. 10.3835/plantgenome2012.03.0002

[11] Mohammadi M, Blake TK, Budde AD, Chao S, Hayes PM, Horsley RD, et al A genome-wide association study of malting quality across eight US barley breeding programs. Theor Appl Genet. 2015;128(4):705–21. 10.1007/s00122-015-2465-5 25666272

[12] Elshire RJ, Glaubitz JC, Sun Q, Poland JA, Kawamoto K, Buckler ES, et al A Robust, Simple Genotyping-by-Sequencing (GBS) Approach for High Diversity Species. PLoS One. 2011;6(5):e19379 10.1371/journal.pone.0019379 21573248 PMC3087801

[13] Poland JA, Brown PJ, Sorrells ME, Jannink JL. Development of High-Density Genetic Maps for Barley and Wheat Using a Novel Two-Enzyme Genotyping-by-Sequencing Approach. PLoS One. 2012;7(2):e32253 10.1371/journal.pone.0032253 22389690 PMC3289635

[14] Close TJ, Bhat PR, Lonardi S, Wu Y, Rostoks N, Ramsay L, et al Development and implementation of high-throughput SNP genotyping in barley. BMC Genomics. 2009;10(1):582. 19961604 10.1186/1471-2164-10-582 PMC2797026

[15] Muñoz-Amatriaín M, Cuesta-Marcos A, Endelman JB, Comadran J, Bonman JM, Bockelman HE, et al The USDA barley core collection: genetic diversity, population structure, and potential for genome-wide association studies. PLoS One. 2014;9(4):e94688 10.1371/journal.pone.0094688 24732668 PMC3986206

[16] Bayer MM, Rapazote-Flores P, Ganal M, Hedley PE, Macaulay M, Plieske J, et al Development and evaluation of a barley 50k iSelect SNP array. Front. Plant Sci. 2017;8:1792 10.3389/fpls.2017.01792 29089957 PMC5651081

[17] Igartua E, Moralejo M, Casas AM, Torres L, Molina-Cano JL. Whole-genome analysis with SNPs from BOPA1 shows clearly defined groupings of Western Mediterranean, Ethiopian, and Fertile Crescent barleys. Genet Resour Crop Ev. 2013;60(1):251–64. 10.1007/s10722-012-9831-9

[18] Turuspekov Y, Abugalieva S, Ermekbayev K, Sato K. Genetic characterization of wild barley populations (Hordeum vulgare ssp. spontaneum) from Kazakhstan based on genome wide SNP analysis. Breeding Science. 2014;64:399–403. 10.1270/jsbbs.64.399 25914595 PMC4267315

[19] Saade S, Maurer A, Shahid M, Oakey H, Schmöckel SM, Negrão S, et al Yield-related salinity tolerance traits identified in a nested association mapping (NAM) population of wild barley. Scientific reports. 2016;6:32586 10.1038/srep32586 27585856 PMC5009332

[20] Wang M, Jiang N, Jia T, Leach L, Cockram J, Comadran J, et al Genome-wide association mapping of agronomic and morphologic traits in highly structured populations of barley cultivars. Theor Appl Genet. 2012;124(2):233–46. 10.1007/s00122-011-1697-2 21915710

[21] Turuspekov Y, Ormanbekova D, Rsaliev A, Abugalieva S. Genome-wide association study on stem rust resistance in Kazakh spring barley lines. BMC Plant Biol. 2016;16(1):6 10.1186/s12870-015-0686-z 26821649 PMC4895317

[22] Xu X, Sharma R, Tondelli A, Russell J, Comadran J, Schnaithmann F, et al Genome-Wide Association Analysis of Grain Yield-Associated Traits in a Pan-European Barley Cultivar Collection. The Plant Genome. 2018;11(1):170073 10.3835/plantgenome2017.08.0073 29505630 PMC12962562

[23] Genievskaya Y, Almerekova S, Sariev B, Chudinov V, Tokhetova L, Sereda G, et al Marker-trait associations in two-rowed spring barley accessions from Kazakhstan and the USA. PLoS One. 2018;13(10): e0205421 10.1371/journal.pone.0205421 30308008 PMC6181366

[24] Berger GL, Liu S, Hall MD, Brooks WS, Chao S, Muehlbauer GJ, et al Marker-trait associations in Virginia Tech winter barley identified using genome-wide mapping. Theor Appl Genet. 2013;126(3):693–710. 10.1007/s00122-012-2011-7 23139143

[25] Alqudah AM, Sharma R, Pasam RK, Graner A, Kilian B, Schnurbusch T. Genetic dissection of photoperiod response based on GWAS of pre-anthesis phase duration in spring barley. PLoS One. 2014;9(11):e113120 10.1371/journal.pone.0113120 25420105 PMC4242610

[26] Bellucci A, Tondelli A, Fangel JU, Torp AM, Xu X, Willats WGT, et al Genome-wide association mapping in winter barley for grain yieldand culm cell wall polymer content using the high-throughput CoMPP technique. PLoS One. 2017;12(3):e0173313 10.1371/journal.pone.0173313 28301509 PMC5354286

[27] Sharma R, Draicchio F, Bull H, Herzig P, Maurer A, Pillen K, et al Genome-wide association of yield traits in a nested association mapping population of barley reveals new gene diversity for future breeding. Journal of Experimental Botany. 2018;69(16): 3811–22. 10.1093/jxb/ery178 29767798 PMC6054221

[28] Hu X, Zuo J, Wang J, Liu L, Sun G, Li C, et al Multi-Locus Genome-Wide Association Studies for 14 Main Agronomic Traits in Barley. Front. Plant Sci. 2018;9:1683 10.3389/fpls.2018.01683 30524459 PMC6257129

[29] Pswarayi A, van Eeuwijk FA, Ceccarelli S, Grando S, Comadran J, Russell JR, et al Changes in allele frequencies in landraces, old and modern barley cultivars of marker loci close to QTL for grain yield under high and low input conditions. Euphytica. 2008;163:435–44. 10.1007/s10681-008-9726-1references

[30] Pasam RK, Sharma R, Malosetti M, van Eeuwijk FA, Haseneyer G, Kilian B, et al Genome-wide association studies for agronomical traits in a world wide spring barley collection. BMC Plant Biol. 2012;12:16 10.1186/1471-2229-12-16 22284310 PMC3349577

[31] Pauli D, Muehlbauer GJ, Smith KP, Cooper B, Hole D, Obert DE, et al Association mapping of agronomic QTLs in US spring barley breeding germplasm. The Plant Genome. 2014;7(3). 10.3835/plantgenome2013.11.0037

[32] Rozas J, Ferrer-Mata A, Sánchez-DelBarrio JC, Guirao-Rico S, Librado P, Ramos-Onsins SE, et al DnaSP 6: DNA sequence polymorphism analysis of large data sets. Mol Biol Evol. 2017;34(12):3299–3302. 10.1093/molbev/msx248 29029172

[33] Peakall R, Smouse PE. GenAlEx 6.5: genetic analysis in Excel. Population genetic software for teaching and research-an update. Bioinformatics. 2012;28:2537–39. 10.1093/bioinformatics/bts460 22820204 PMC3463245

[34] Falush D, Stephans M, Pritchard JK. Inference of population structure using multilocus genotype data: linked loci and correlated allele frequencies. Genetics. 2003;164:1567–1587. 12930761 10.1093/genetics/164.4.1567 PMC1462648

[35] Evanno G, Regnaut S, Goudet J. Detecting the number of clusters of individuals using the software STRUCTURE: a simulation study. Mol Ecol. 2005;14(8):2611–20. 10.1111/j.1365-294X.2005.02553.x 15969739

[36] Earl DA, von Holdt BM. STRUCTURE HARVESTER: a website and program for visualizing STRUCTURE output and implementing the Evanno method. Conserv. Genet. Resour. 2012;4(2):359–61.

[37] Bradbury PJ, Zhang Z, Kroon DE, Casstevens TM, Ramdoss Y, Buckler ES. TASSEL: Software for association mapping of complex traits in diverse samples. Bioinformatics 2007;23:2633–35. 10.1093/bioinformatics/btm308 17586829

[38] Yu J, Pressoir G, Briggs WH, Bi IV, Yamasaki M, Doebley JF, et al A unified mixed-model method for association mapping that accounts for multiple levels of relatedness. Nat. Genet. 2006;38:203–8. 10.1038/ng1702 16380716

[39] Korte A, Vilhjálmsson BJ, Segura V, Platt A, Long Q, Nordborg M. A mixed-model approach for genome-wide association studies of correlated traits in structured populations. Nat. Genet. 2012;44(9):1066–71. 10.1038/ng.2376 22902788 PMC3432668

[40] Voorrips RE. MapChart: software for the graphical presentation of linkage maps and QTLs. J. Hered. 2002;93:77–8. 10.1093/jhered/93.1.77 12011185

[41] IBSC. High-resolution GBS map of the Morex x Barke RIL population. 2016. 10.5447/ipk/2016/29.

[42] Rode J, Ahlemeyer J, Friedt W, Ordon F. Identification of marker-trait associations in the German winter barley breeding gene pool (Hordeum vulgare L.). Mol Breed. 2012;30(2):831–43.

[43] Cockram J, Thiel T, Steuernagel B, Stein N, Taudien S, Bailey PC, et al Genome dynamics explain the evolution of flowering time CCT domain gene families in the Poaceae. PLoS One. 2012;7:e45307 10.1371/journal.pone.0045307 23028921 PMC3454399

[44] Casao MC, Igartua E, Karsai I, Lasa JM, Gracia MP, Casas AM, et al Expression analysis of vernalization and day-length response genes in barley (Hordeum vulgare L.) indicates that VRNH2 is a repressor of PPDH2 (HvFT3) under long days. Journal of Experimental Botany. 2011;62:1939–49 10.1093/jxb/erq382 21131547 PMC3060678

[45] Wang G, Schmalenbach I, von Korff M, Léon J, Kilian B, Rode J, et al Association of barley photoperiod and vernalization genes with QTLs for flowering time and agronomic traits in a BC 2 DH population and a set of wild barley introgression lines. Theoretical and Applied Genetics. 2010;120(8):1559–74. 10.1007/s00122-010-1276-y 20155245 PMC2859222

[46] Takahashi R, Yamamoto J. Studies on the Classification and the Geographical Distribution of the Japanese Barley Varieties. III On the Linkage Relation and the Origin of the ‘uzu’ or Semi-Brachytic Character in Barley. Berichte des Ohara Instituts für landwirtschaftliche Forschungen. 1951;9(4):399–410.

[47] Franckowiak JD. Revised linkage maps for morphological markers in barley, *Hordeum vulgare*. Barley Genetics Newsletter. 1997;26:9–21.

[48] Costa JM, Corey A, Hayes PM, Jobet C, Kleinhofs A, Kopisch-Obusch A, et al Molecular mapping of the Oregon Wolfe Barleys: a phenotypically polymorphic doubled-haploid population. Theoretical and Applied Genetics. 2001;103(2–3):415–24.

[49] Turuspekov Y, Kawada N, Honda I, Watanabe Y, Komatsuda T. Identification and mapping of a QTL for rachis internode length associated with cleistogamy in barley. Plant Breeding. 2005;124(6):542–45. 10.1111/j.1439-0523.2005.01161.x

[50] Sameri M, Takeda K, Komatsuda T. Quantitative trait loci controlling agronomic traits in recombinant inbred lines from a cross of oriental- and occidental-type barley cultivars. Breed. Sci. 2006;56(3):243–52. 10.1270/jsbbs.56.243

[51] Reynolds MP, Trethowan R, Crossa J, Vargas M, Sayre KD. Physiological factors associated with genotype by environment interaction in wheat. Field Crops Res. 2002;75:139–60. 10.1016/S0378-4290(02)00023-0

[52] Mangini G, Gadaleta A, Colasuonno P, Marcotuli I, Signorile AM, Simeone R, et al Genetic dissection of the relationships between grain yield components by genome-wide association mapping in a collection of tetraploid wheats. PLoS One. 2018;13(1):e0190162 10.1371/journal.pone.0190162 29324803 PMC5764242

[53] Semagn K, Babu R, Hearne S, Olsen M. Single nucleotide polymorphism genotyping using Kompetitive Allele Specific PCR (KASP): overview of the technology and its application in crop improvement. Mol. Breeding. 2014;33:1–14.

