# Supplementary material for: Association mapping for agronomic traits in six-rowed spring barley from the USA harvested in Kazakhstan
Source: PLoS One. 2019 Aug 12;14(8):e0221064. doi: 10.1371/journal.pone.0221064 (PMC6690582; doi:10.1371/journal.pone.0221064)
Supplement: S1 Appendix — A–AK (Aktobe), B–AL (Almaty), C–KA (Karaganda), D–KB (Kostanai), E–KO (Kyzylorda), F–KV (South Kazakhstan). (PDF) [file pone.0221064.s001.pdf]

GWAS RESULTS

The distribution lines of the QQ plots for 6 studied traits in six breeding programs

A – AK (Aktobe), B – AL (Almaty), C – KA (Karaganda),
D – KB (Kostanai), E – KO (Kyzylorda), F – KV (South Kazakhstan).

HT

A

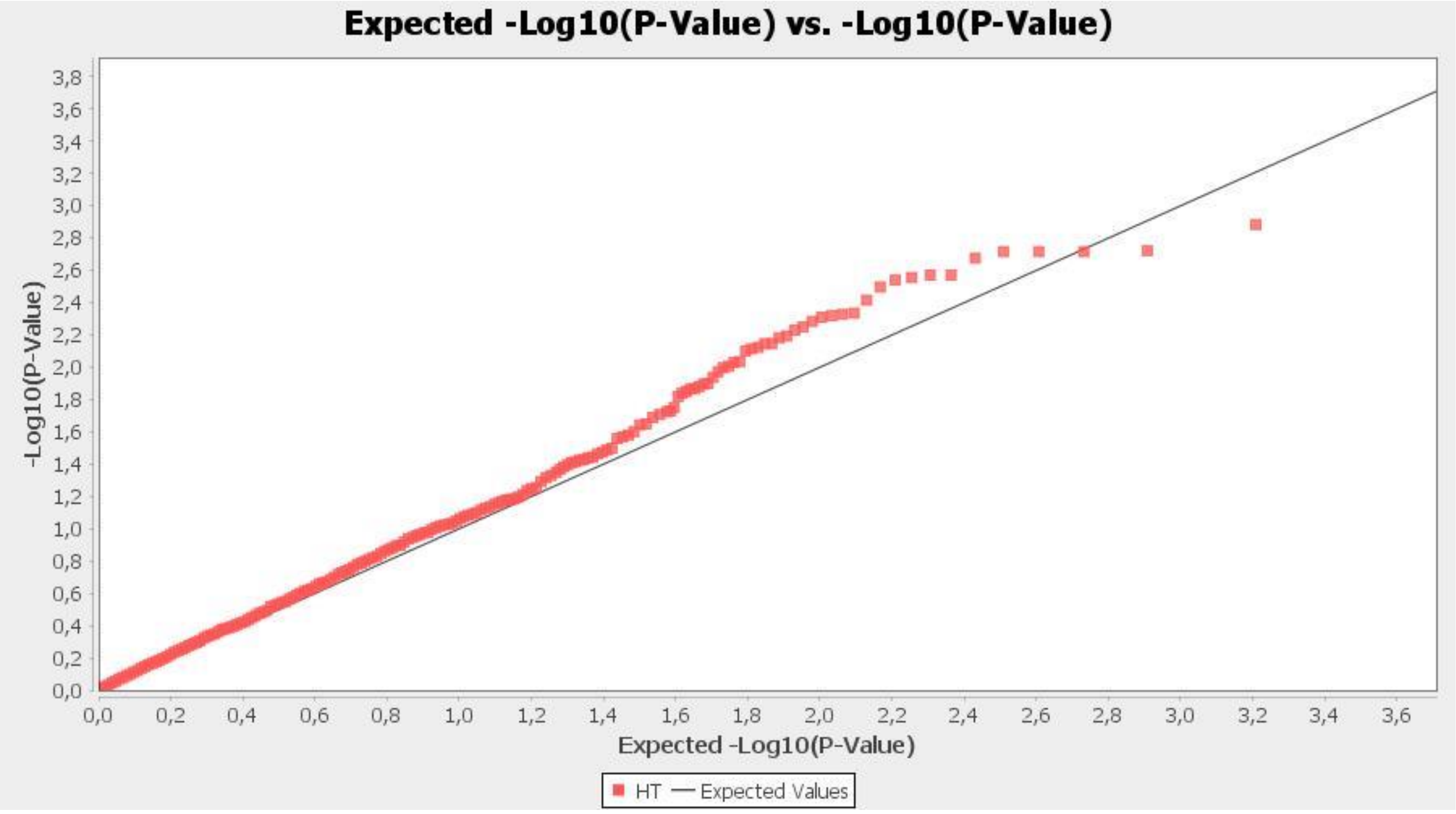
Expected -Log10(P-Value) vs. -Log10(P-Value)
-Log10(P-Value)
Expected -Log10(P-Value)
HT — Expected Values

B

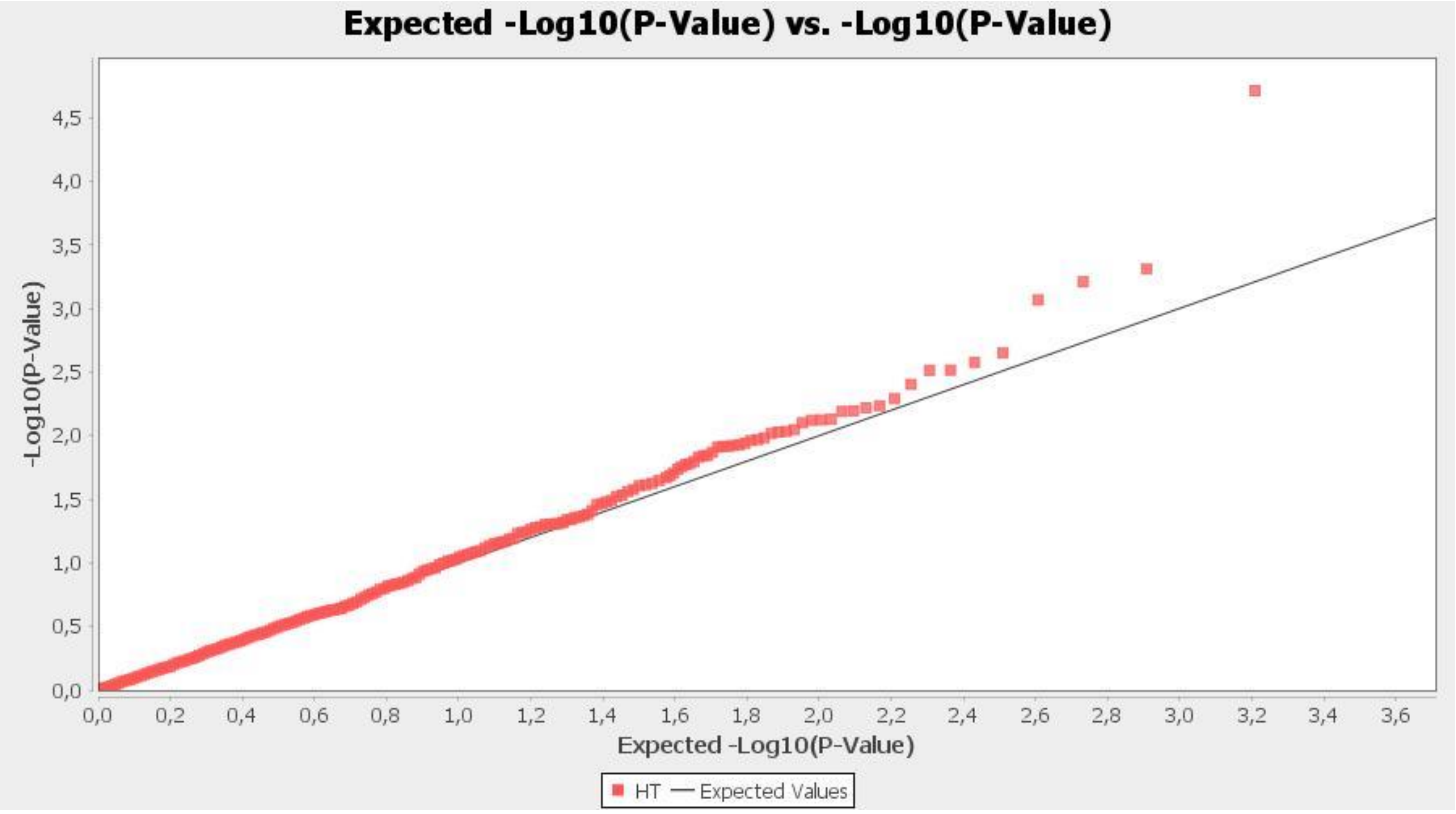
Expected -Log10(P-Value) vs. -Log10(P-Value)
-Log10(P-Value)
Expected -Log10(P-Value)
HT — Expected Values

C

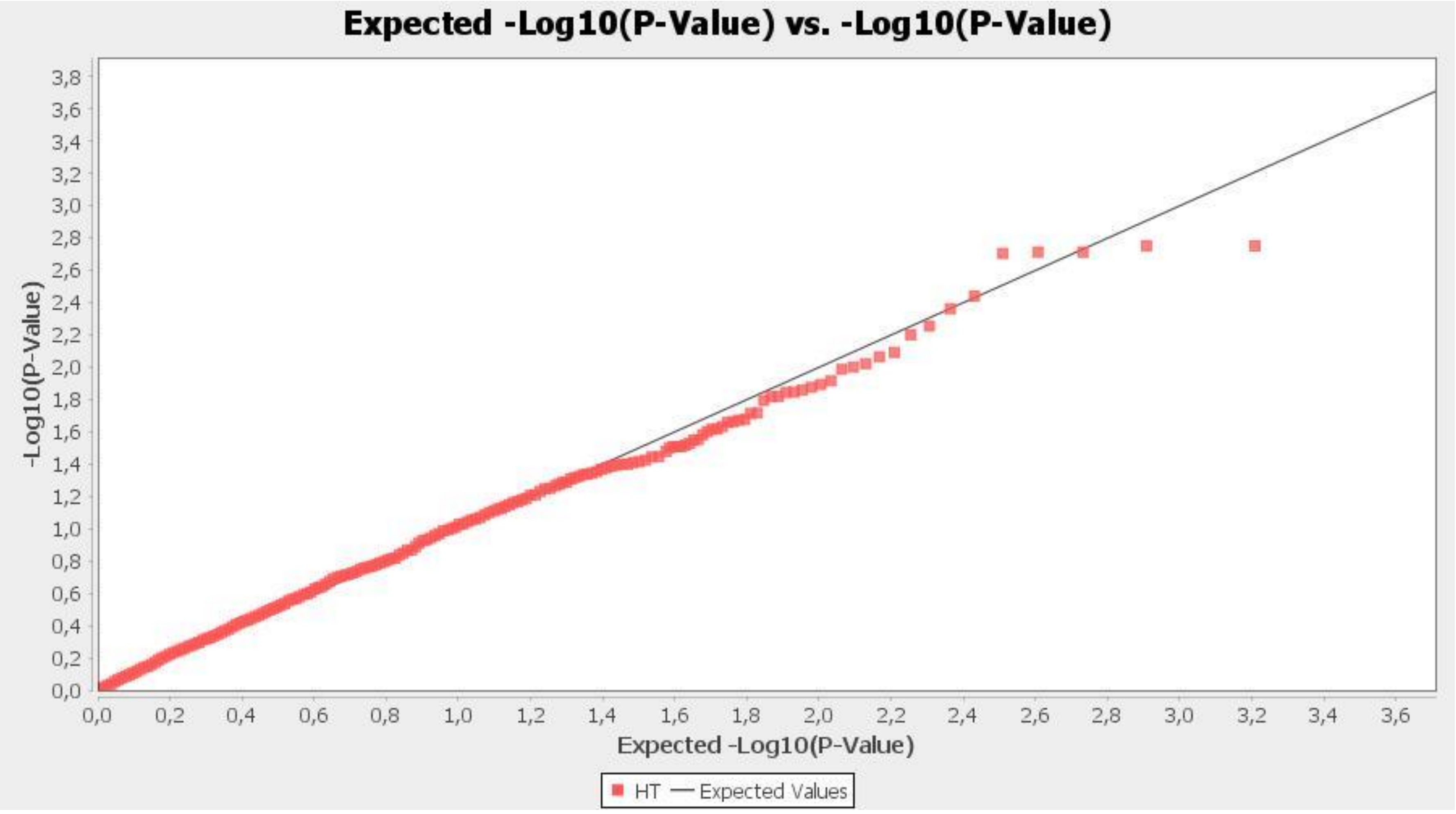
Expected -Log10(P-Value) vs. -Log10(P-Value)
-Log10(P-Value)
Expected -Log10(P-Value)
HT — Expected Values

D

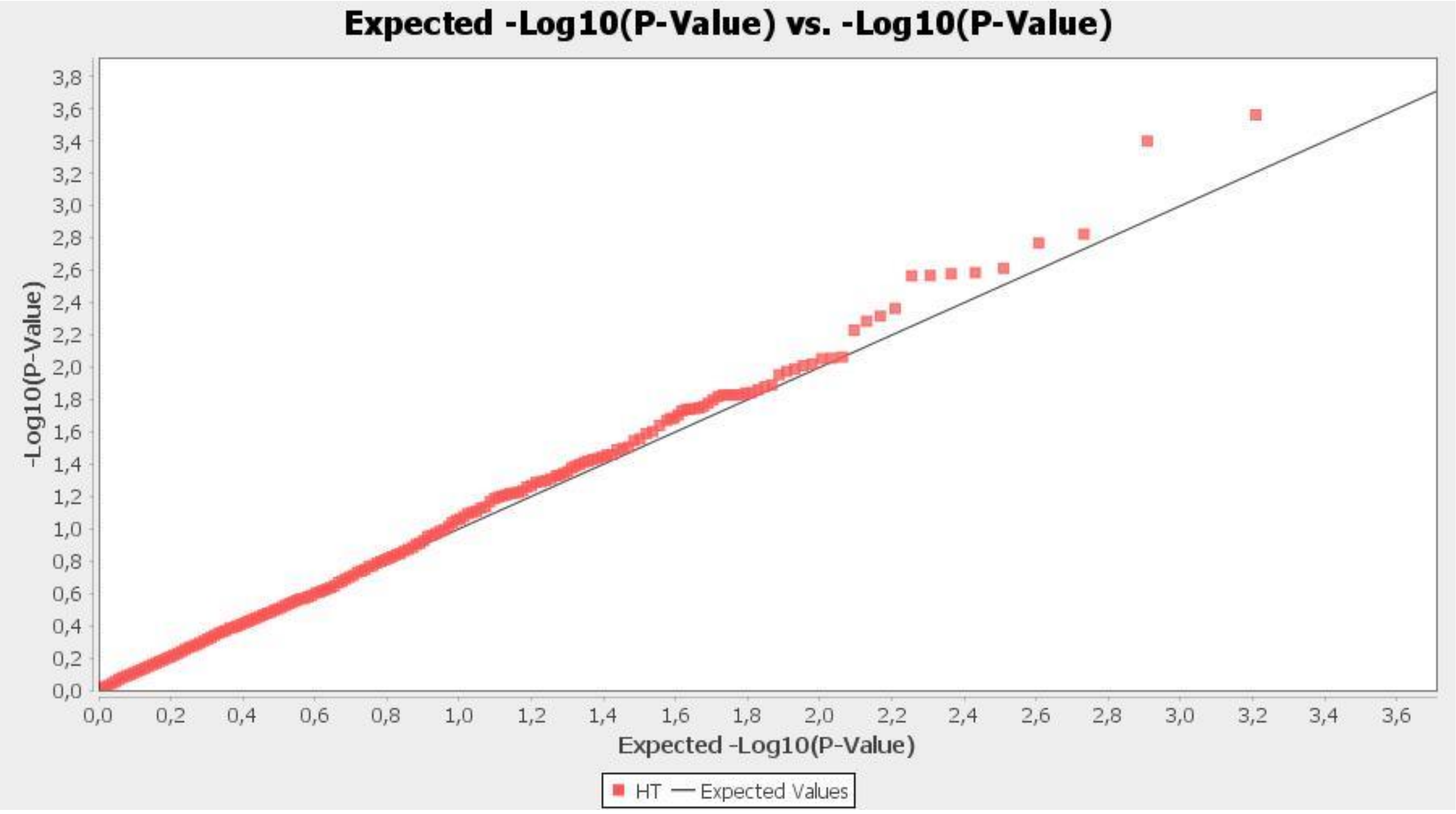
Expected -Log10(P-Value) vs. -Log10(P-Value)
-Log10(P-Value)
Expected -Log10(P-Value)
HT — Expected Values

E

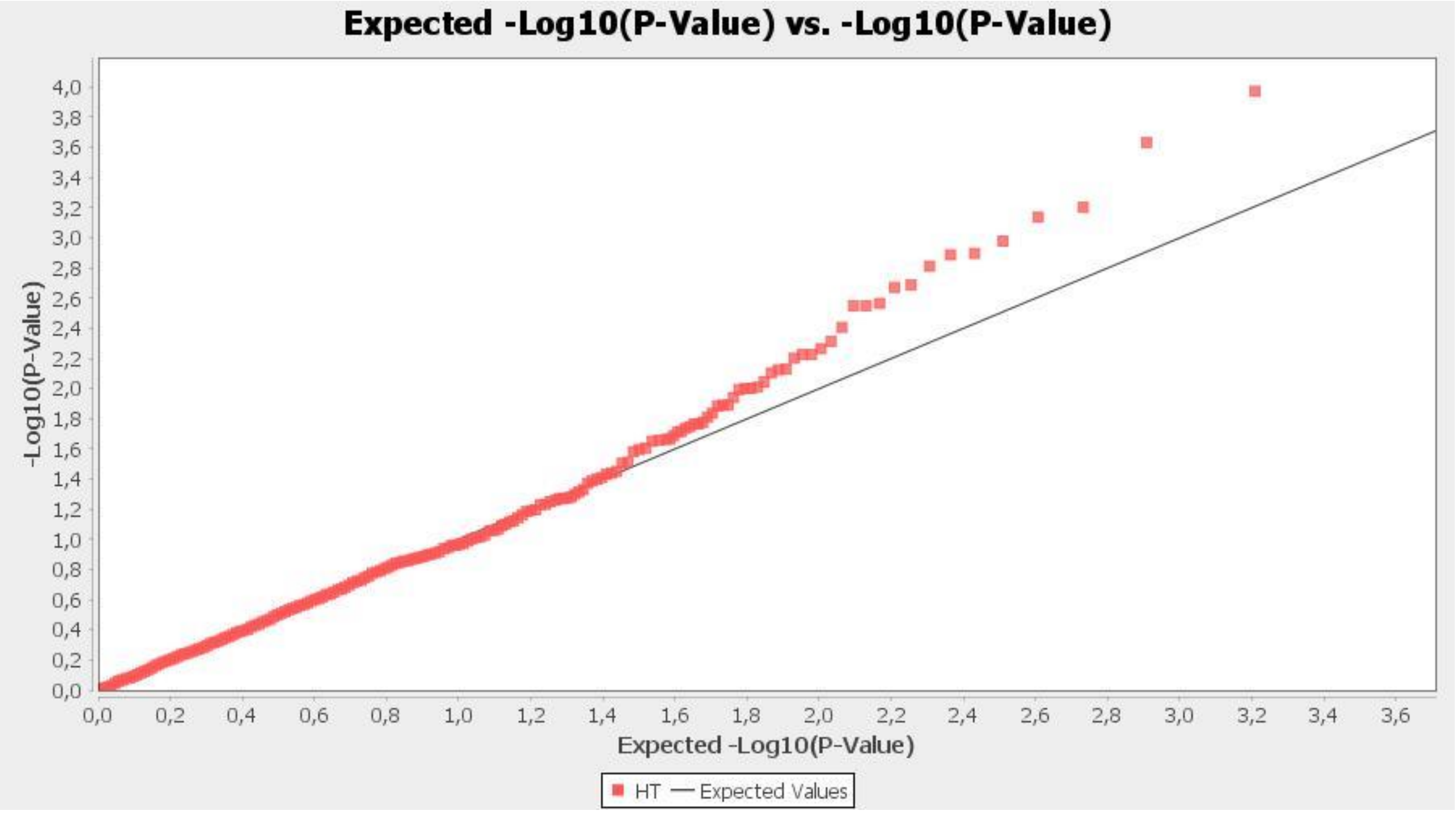
Expected -Log10(P-Value) vs. -Log10(P-Value)
-Log10(P-Value)
Expected -Log10(P-Value)
HT — Expected Values

F

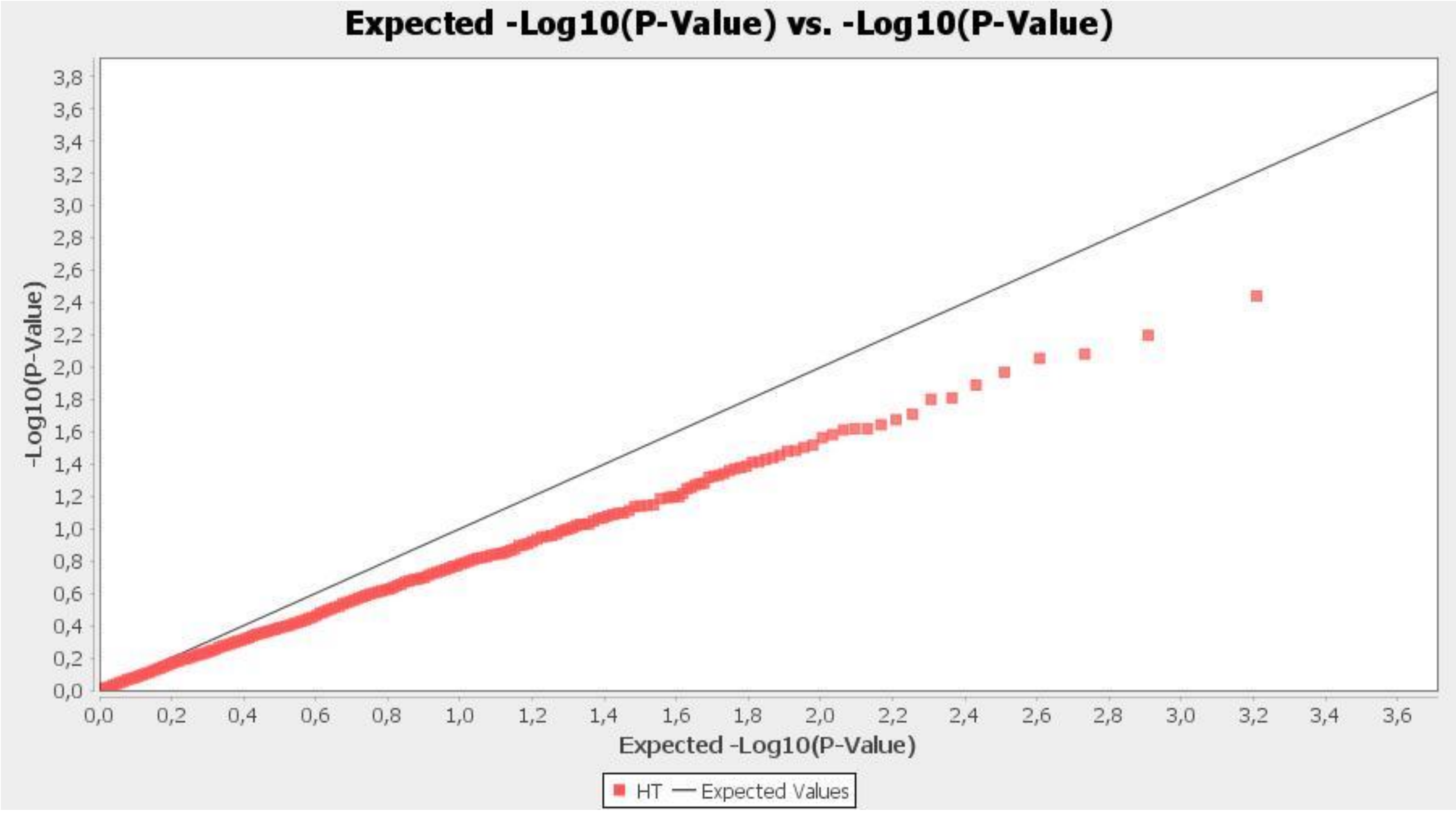
Expected -Log10(P-Value) vs. -Log10(P-Value)
-Log10(P-Value)
Expected -Log10(P-Value)
HT — Expected Values

SMT

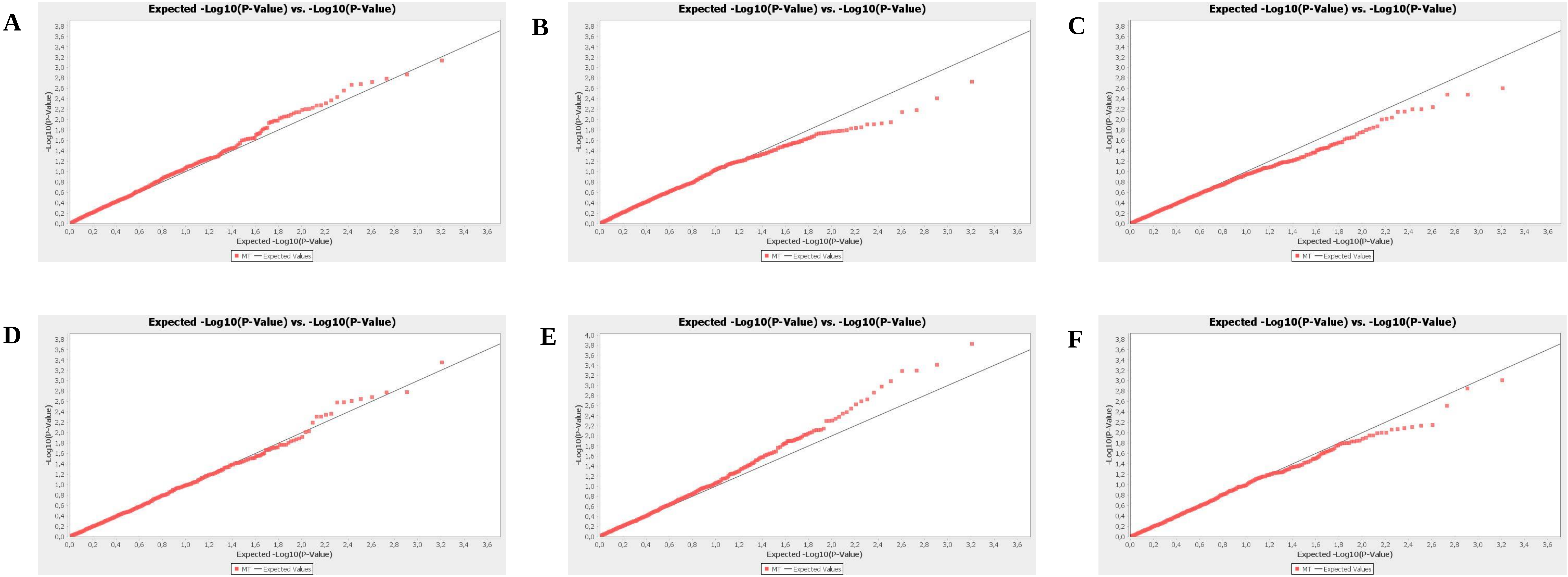
A
B
C
D
E
F
Expected -Log10(P-Value) vs. -Log10(P-Value)
-Log10(P-Value)
Expected -Log10(P-Value)
MT — Expected Values

PH

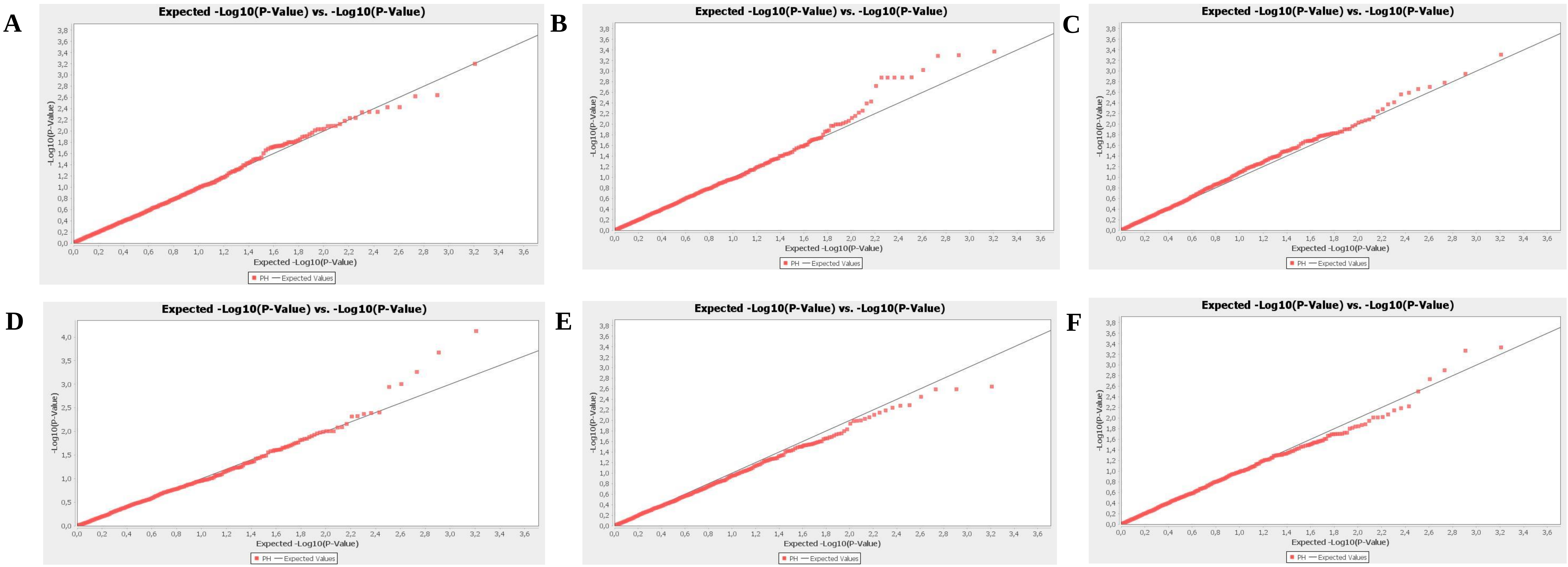
A
Expected -Log10(P-Value) vs. -Log10(P-Value)
-Log10(P-Value)
Expected -Log10(P-Value)
PH — Expected Values
B
Expected -Log10(P-Value) vs. -Log10(P-Value)
-Log10(P-Value)
Expected -Log10(P-Value)
PH — Expected Values
C
Expected -Log10(P-Value) vs. -Log10(P-Value)
-Log10(P-Value)
Expected -Log10(P-Value)
PH — Expected Values
D
Expected -Log10(P-Value) vs. -Log10(P-Value)
-Log10(P-Value)
Expected -Log10(P-Value)
PH — Expected Values
E
Expected -Log10(P-Value) vs. -Log10(P-Value)
-Log10(P-Value)
Expected -Log10(P-Value)
PH — Expected Values
F
Expected -Log10(P-Value) vs. -Log10(P-Value)
-Log10(P-Value)
Expected -Log10(P-Value)
PH — Expected Values

NKS

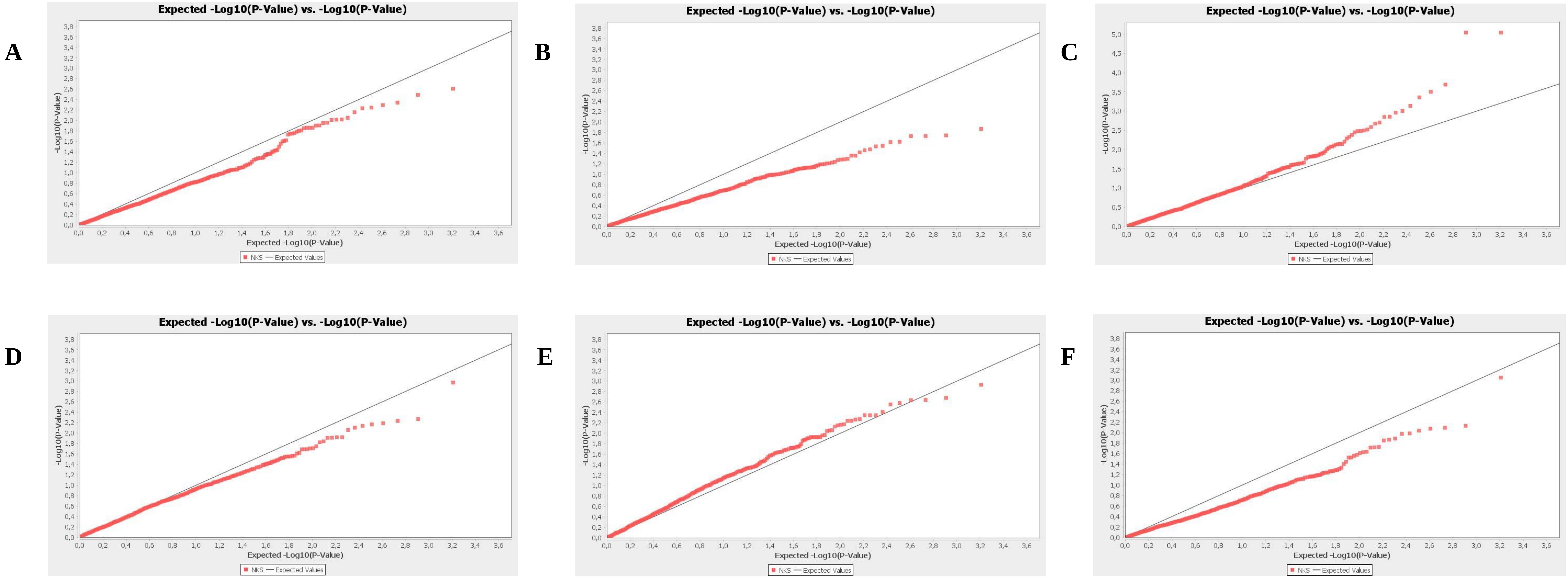
A
Expected -Log10(P-Value) vs. -Log10(P-Value)
-Log10(P-Value)
Expected -Log10(P-Value)
NKS — Expected Values
B
Expected -Log10(P-Value) vs. -Log10(P-Value)
-Log10(P-Value)
Expected -Log10(P-Value)
NKS — Expected Values
C
Expected -Log10(P-Value) vs. -Log10(P-Value)
-Log10(P-Value)
Expected -Log10(P-Value)
NKS — Expected Values
D
Expected -Log10(P-Value) vs. -Log10(P-Value)
-Log10(P-Value)
Expected -Log10(P-Value)
NKS — Expected Values
E
Expected -Log10(P-Value) vs. -Log10(P-Value)
-Log10(P-Value)
Expected -Log10(P-Value)
NKS — Expected Values
F
Expected -Log10(P-Value) vs. -Log10(P-Value)
-Log10(P-Value)
Expected -Log10(P-Value)
NKS — Expected Values

TGW

A

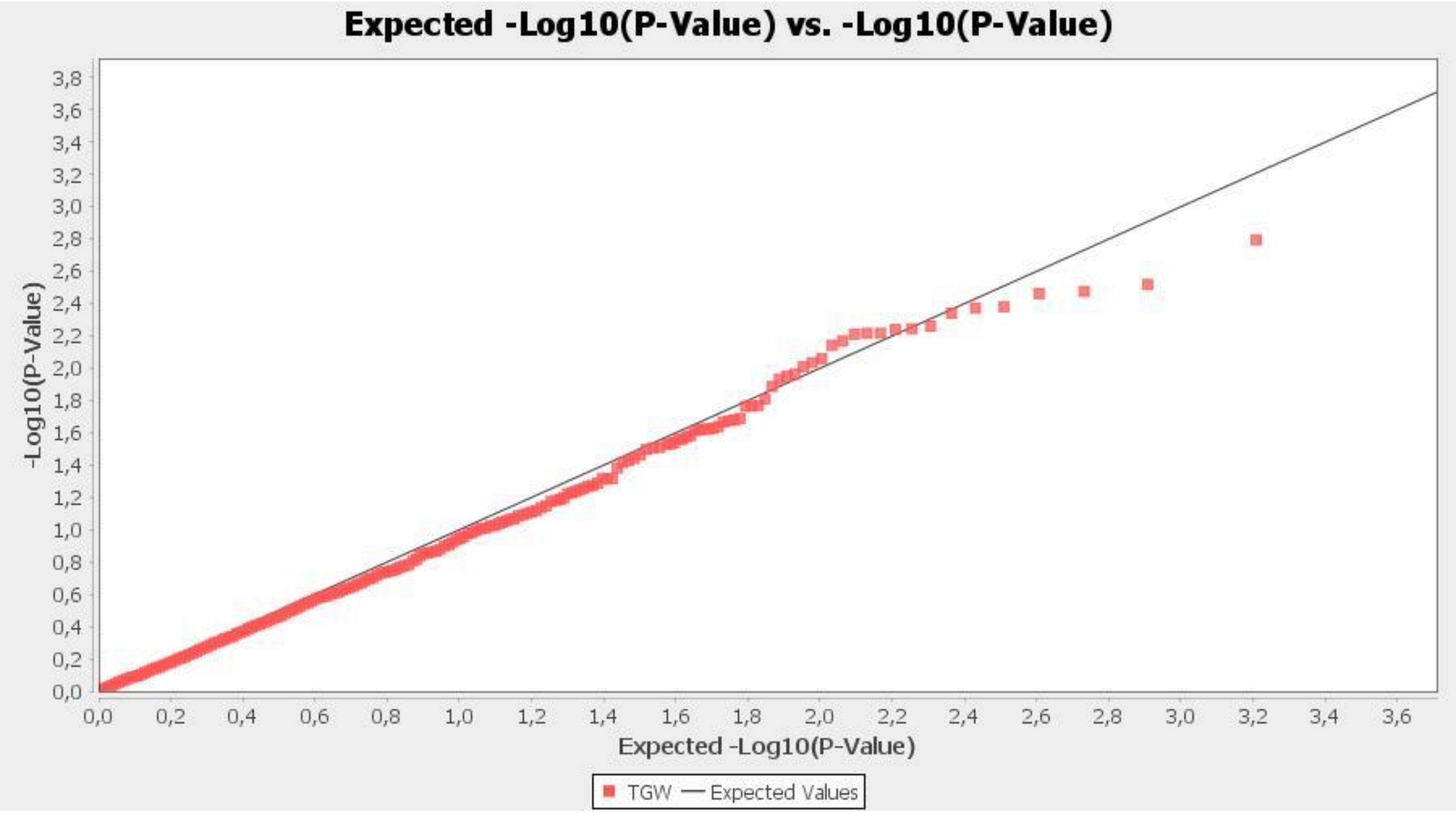
Expected -Log10(P-Value) vs. -Log10(P-Value)
-Log10(P-Value)
Expected -Log10(P-Value)
TGW — Expected Values

B

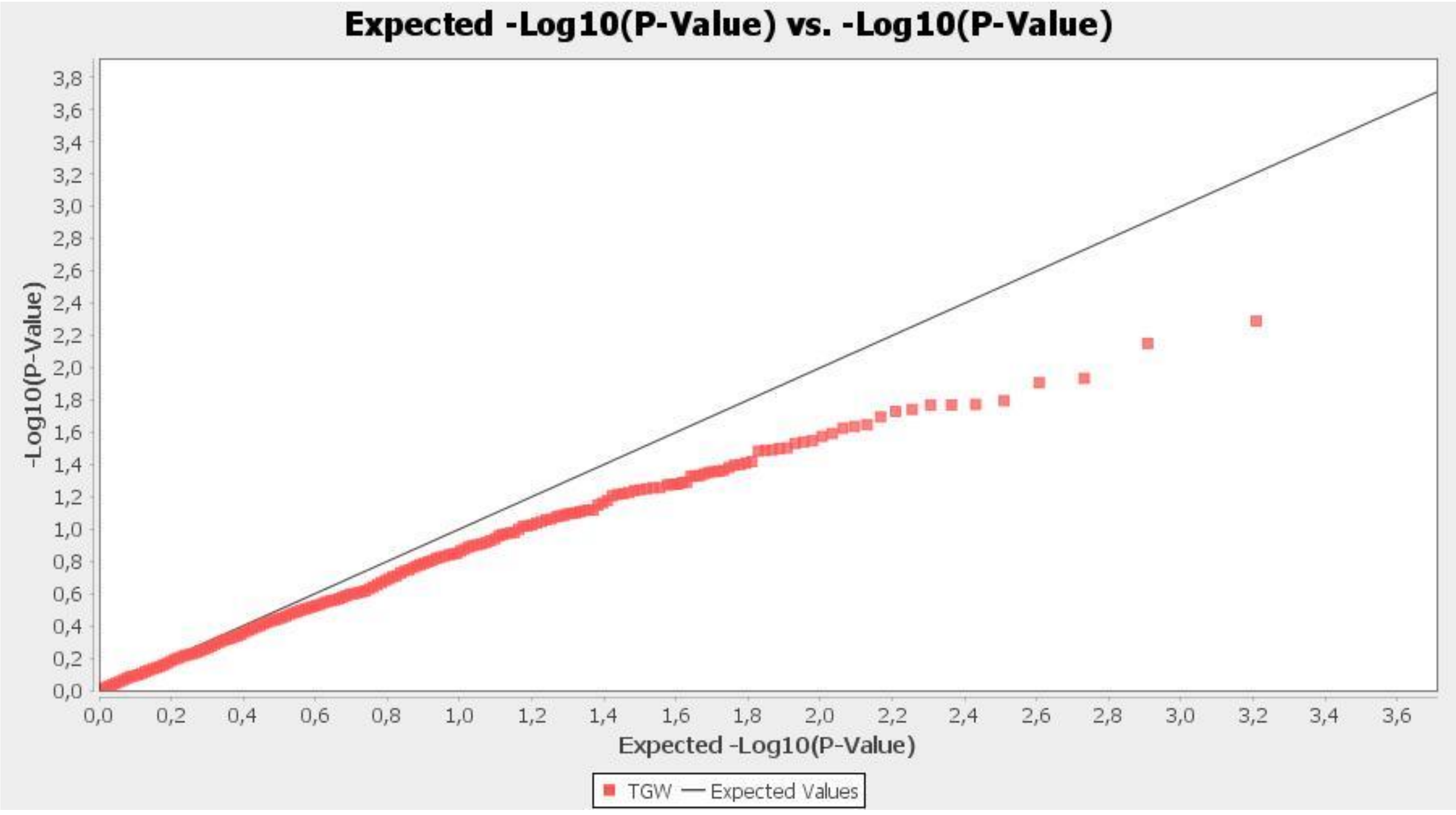
Expected -Log10(P-Value) vs. -Log10(P-Value)
-Log10(P-Value)
Expected -Log10(P-Value)
TGW — Expected Values

C

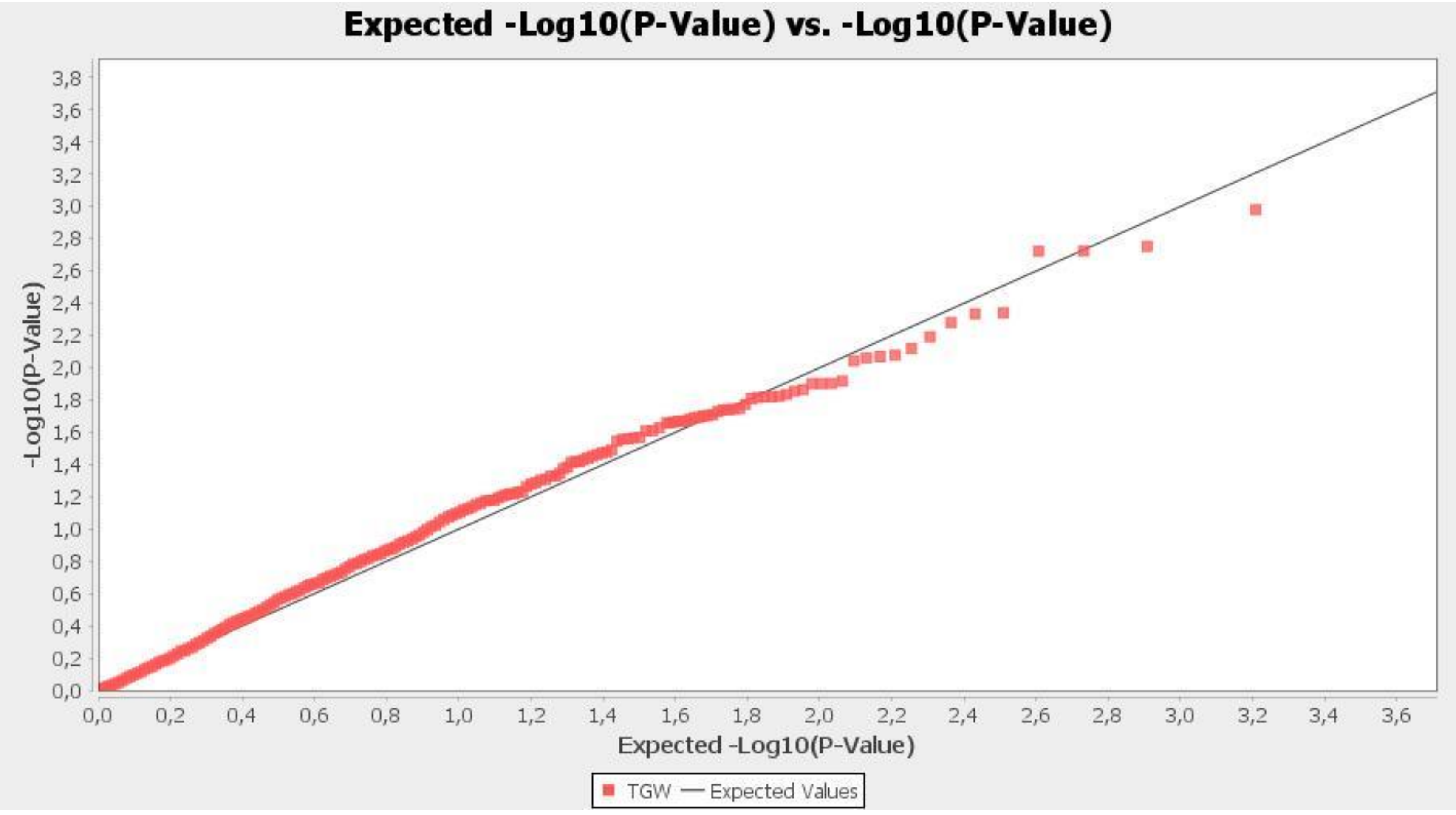
Expected -Log10(P-Value) vs. -Log10(P-Value)
-Log10(P-Value)
Expected -Log10(P-Value)
TGW — Expected Values

D

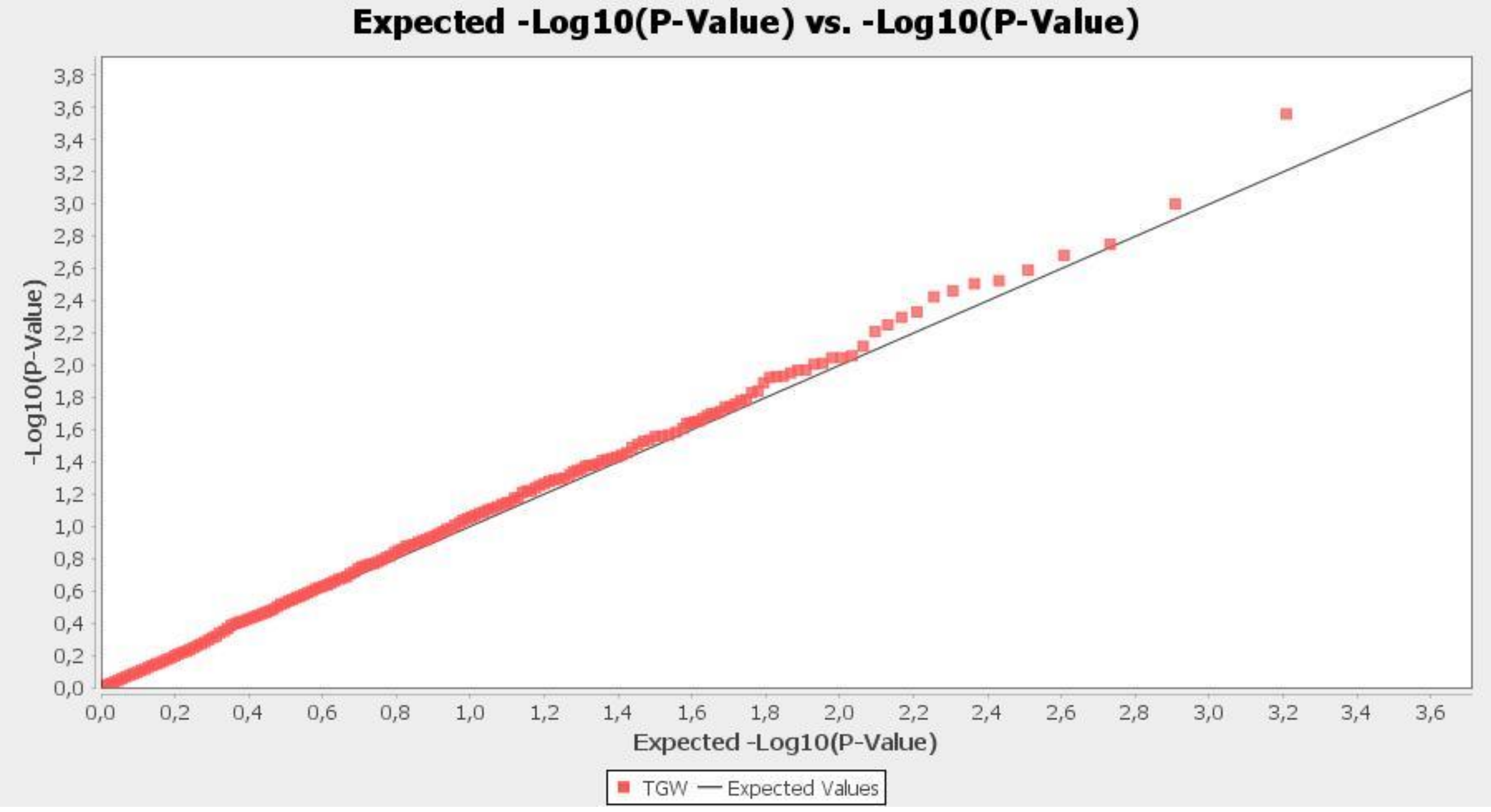
Expected -Log10(P-Value) vs. -Log10(P-Value)
-Log10(P-Value)
Expected -Log10(P-Value)
TGW — Expected Values

E

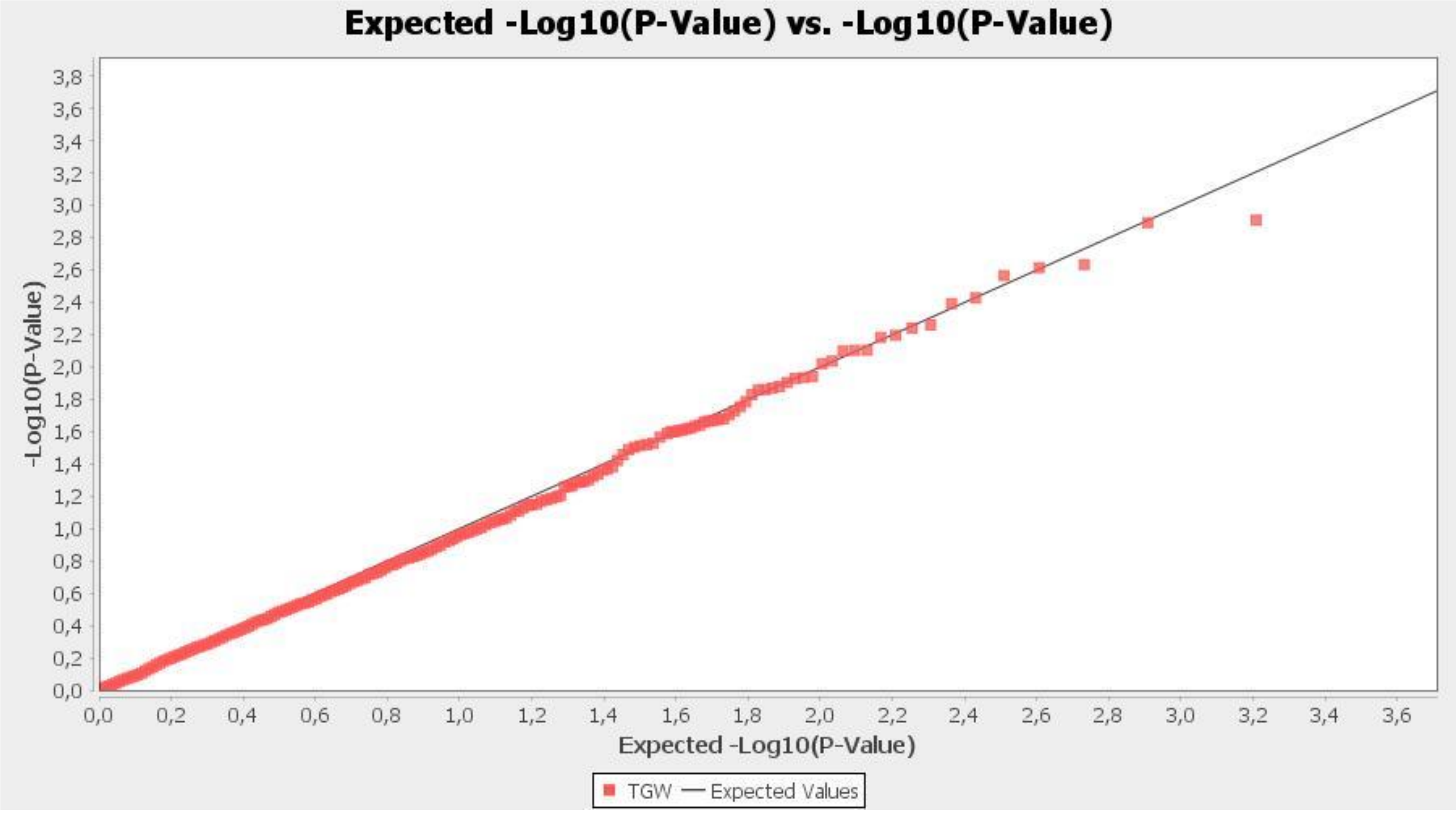
Expected -Log10(P-Value) vs. -Log10(P-Value)
-Log10(P-Value)
Expected -Log10(P-Value)
TGW — Expected Values

F

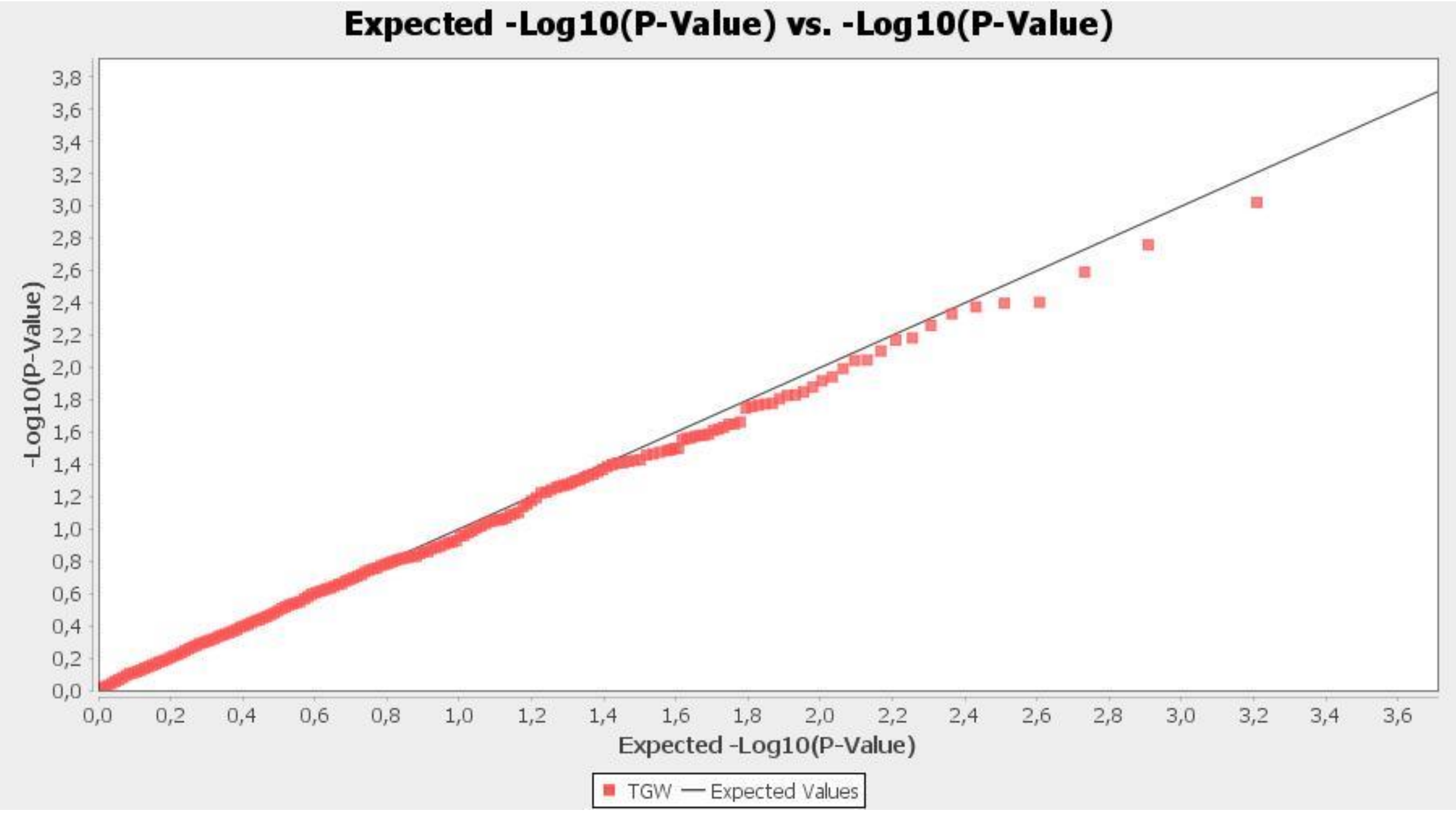
Expected -Log10(P-Value) vs. -Log10(P-Value)
-Log10(P-Value)
Expected -Log10(P-Value)
TGW — Expected Values

YM2

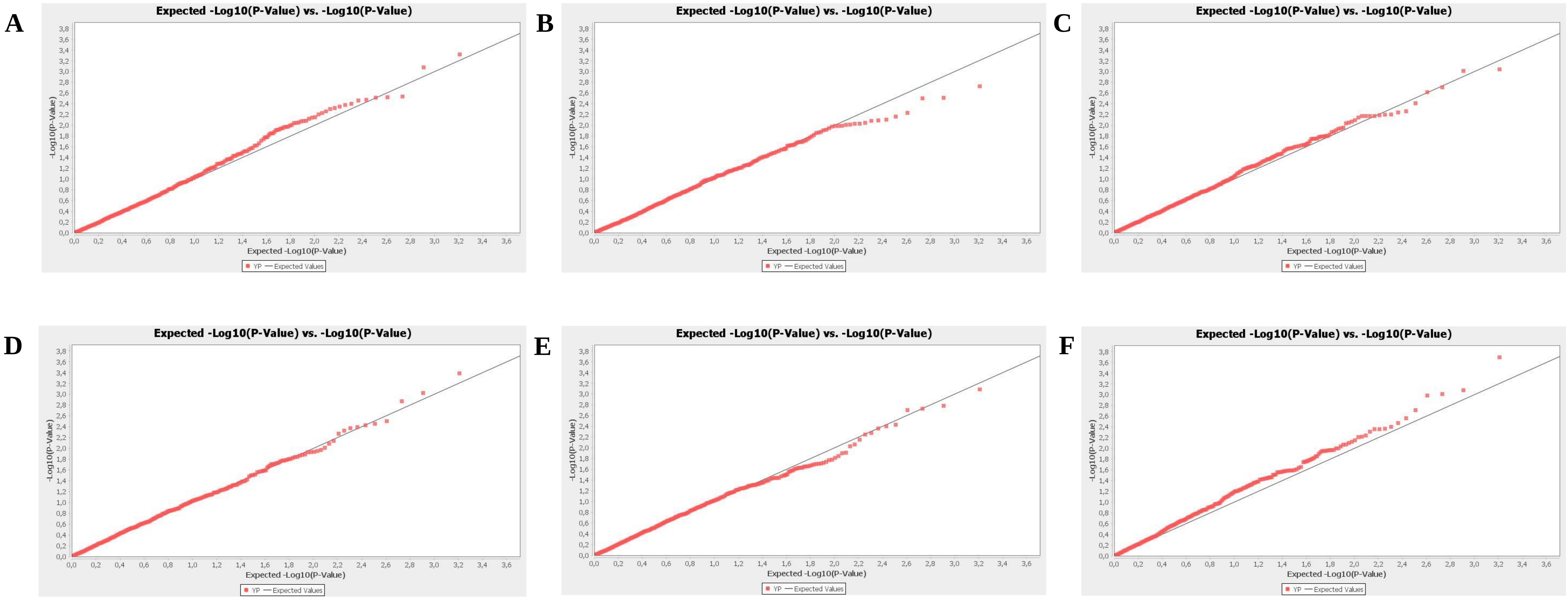
A
Expected -Log10(P-Value) vs. -Log10(P-Value)
-Log10(P-Value)
Expected -Log10(P-Value)
YP — Expected Values
B
Expected -Log10(P-Value) vs. -Log10(P-Value)
-Log10(P-Value)
Expected -Log10(P-Value)
YP — Expected Values
C
Expected -Log10(P-Value) vs. -Log10(P-Value)
-Log10(P-Value)
Expected -Log10(P-Value)
YP — Expected Values
D
Expected -Log10(P-Value) vs. -Log10(P-Value)
-Log10(P-Value)
Expected -Log10(P-Value)
YP — Expected Values
E
Expected -Log10(P-Value) vs. -Log10(P-Value)
-Log10(P-Value)
Expected -Log10(P-Value)
YP — Expected Values
F
Expected -Log10(P-Value) vs. -Log10(P-Value)
-Log10(P-Value)
Expected -Log10(P-Value)
YP — Expected Values
