# Supplementary material for: Association mapping for agronomic traits in six-rowed spring barley from the USA harvested in Kazakhstan
Source: PLoS One. 2019 Aug 12;14(8):e0221064. doi: 10.1371/journal.pone.0221064 (PMC6690582; doi:10.1371/journal.pone.0221064)
Supplement: S3 Table — (PDF) [file pone.0221064.s004.pdf]

S3 Table. Comparison of identified MTA for six-rowed barley in this study to previously reported genetic factors related to ten analyzed traits

Current study				Previous studies				
**Trait**	**Marker ID**	**Chr.**	**Pos. (cM)**	**Gene/QTL/ Marker**	**Chr.**	**Pos. (cM)**	**Ref.**	**Gene ID sequence alignment (EnsemblPlants)**
HT	11_10259	1H	48.1	ConsensusGBS0455-1	1H	41.0	[42]	HORVU1Hr1G017700
				HvCMF10	1H	47.8	[43]	
				11_11336	1H	50.0	[23]	
HT	11_21373	1H	90.9	*Ppd-H2 / HvFT3*	1H	92.3	[44]	HORVU1Hr1G074940
				i_SCRI_RS_130139	1H	93.1	[25]	
HT	11_11016	3H	59.6	*QTL8_HD*	3H	52.0	[45]	HORVU3Hr1G062710
				HvGI	3H	59.9	[30]	
				4453-422	3H	60.0	[42]	
				HvFT2	3H	65.5	[46]	
HT	12_30554	4H	95.2	12_30554	4H	96.6	[31]	HORVU4Hr1G081100
HT	11_21521	6H	2.9	-	-	-	-	HORVU6Hr1G000130
HT	11_11122	7H	74.8	*QTL17_HD*	7H	68.5	[30]	HORVU7Hr1G046040
				HVCO15 HvLHY / HvCCA1	7H	70.5-70.8	[43, 47]	
SMT	11_10396	1H	94.5	*Ppd-H2 / HvFT3*	1H	92.3	[44]	HORVU1Hr1G075680
				i_SCRI_RS_130139	1H	93.1	[25]	
SMT	11_10342	2H	55.4	-	-	-	-	-
SMT	12_10218	7H	40.7	-	-	-	-	HORVU7Hr1G024670
NKS	12_30352	2H	157.4	-	-	-	-	HORVU0Hr1G005670
NKS	12_11192	5H	179.9	-	-	-	-	HORVU5Hr1G123770
PH	11_10253	3H	82.2	11_21505	3H	79.1	[23]	HORVU3Hr1G080930
				HvLNT-1	3H	90.2	[48]	
PH	11_10821	3H	113.4	*qtncPH-3H-1*	3H	631870705-633068955*	[28]	HORVU3Hr1G090760
PH	12_31357	7H	63.4	-	-	-	-	HORVU7Hr1G033370
PH	12_20217	7H	122.6	*qtnPH-7H-3*	7H	622802079*	[28]	HORVU7Hr1G107740
				HvESR1 / HvCO6	7H	120.4-120.8	[48]	
PH	12_30593	7H	150.9	*QTL19_PHT*	7H	140.6	[48]	HORVU7Hr1G116040
				HvBRD2 / HvDIM / HvDWF1	7H	144.5	[30]	
PL	11_11502	3H	58.3	-	-	-	-	HORVU3Hr1G034440
PL	11_21502	3H	64.1	-	-	-	-	HORVU3Hr1G066960

PL	11_10253	3H	82.2	11_21505	3H	79.1	[23]	HORVU3Hr1G080930
PL	11_11375	5H	116.9	11_20188	5H	126.4	[23]	HORVU5Hr1G093450
PT	12_31099	1H	65.8	-	-	-	-	HORVU1Hr1G065150
PT	11_10943	2H	25.5	-	-	-	-	HORVU2Hr1G010990
PT	11_20891	2H	67.9	-	-	-	-	HORVU2Hr1G032400
PT	11_11435	2H	81.3	-	-	-	-	HORVU2Hr1G089020
PT	11_20549	5H	87.7	11_10834	5H	87.7	[23]	HORVU5Hr1G079470
RIL	11_21144	2H	72.4	12_10948	2H	68.8	[23]	HORVU2Hr1G081460
RIL	11_20498	2H	107.5	-	-	-	-	HORVU2Hr1G099470
RIL	11_10926	3H	58.3	*QTL_hotspot_3_1*	3H	52.6-53.5	[22]	HORVU3Hr1G057750
RIL	11_10584	3H	105.9	-	-	-	-	HORVU3Hr1G088270<br>CAQ00109.1 (UniProt)
RIL	11_21065	5H	21.2	-	-	-	-	HORVU5Hr1G005910
RIL	11_20531	6H	102.0	-	-	-	-	HORVU6Hr1G083840<br>HORVU6Hr1G083850
SL	11_10471	1H	83.2	-	-	-	-	HORVU1Hr1G072270
SL	11_11502	3H	58.3	*QTL_hotspot_3_1*	3H	52.6-53.5	[22]	HORVU3Hr1G034440
SL	11_10584	3H	106.0	-	-	-	-	HORVU3Hr1G088270<br>CAQ00109.1 (UniProt)
TGW	11_11435	2H	81.3	*QTL_hotspot_2_2*	2H	78.3-79.9	[22]	HORVU2Hr1G089020
				5644-206	2H	83.0	[42]	
				QTL6_TGW	2H	86.6	[30]	
				Vrs1	2H	92.6	[49]	
TGW	12_30842	6H	19.8	-	-	-	-	HORVU6Hr1G006880
TGW	12_30219	7H	32.9	*QGwe.HaTR-7H.1*	7H	34.8	[30]	HORVU7Hr1G022250
TGW	12_30065	7H	46.7	-	-	-	-	HORVU7Hr1G026940
TGW	12_31357	7H	63.4	-	-	-	-	HORVU7Hr1G033370
TGW	11_10687	7H	146.0	*QTL21_TGW*	7H	143.7	[30]	HORVU7Hr1G114660
YM2	11_21068	1H	128.9	11_21068	1H	128.9	[23]	HORVU1Hr1G087890<br>HORVU1Hr1G087900
YM2	11_11054	2H	64.6	-	-	-	-	HORVU2Hr1G027640
YM2	11_20063	3H	76.17	11_21505	3H	79.1	[23]	HORVU3Hr1G077730
YM2	11_20372	5H	44.99	-	-	-	-	-
YM2	11_21241	5H	127.4	11_20884	5H	126.4	[23]	HORVU5Hr1G096050
YM2	11_20783	6H	92.1	-	-	-	-	HORVU6Hr1G080280
YM2	11_20036	6H	110.6	-	-	-	-	HORVU6Hr1G085830
